# Assay Harmonization and Use of Biological Standards To Improve the Reproducibility of the Hemagglutination Inhibition Assay: a FLUCOP Collaborative Study

**DOI:** 10.1128/mSphere.00567-21

**Published:** 2021-07-28

**Authors:** Joanna Waldock, Lingyi Zheng, Edmond J. Remarque, Alexandre Civet, Branda Hu, Sarah Lartey Jalloh, Rebecca Jane Cox, Sammy Ho, Katja Hoschler, Thierry Ollinger, Claudia Maria Trombetta, Othmar G. Engelhardt, Catherine Caillet

**Affiliations:** a National Institute for Biological Standards and Control, Potters Bar, United Kingdom; b Sanofi Pasteur, Swiftwater, Pennsylvania, USA; c Biomedical Primate Research Centre, Rijswijk, The Netherlands; d Quinten, Paris, France; e Influenza Centre, Department of Clinical Sciences, University of Bergen, Bergen, Norway; f Public Health England, Colindale, United Kingdom; g GSK, Wavre, Belgium; h University of Siena, Department of Molecular and Developmental Medicine, Siena, Italy; University of Maryland School of Medicine

**Keywords:** influenza, standardization, serology, hemagglutination inhibition assay

## Abstract

The hemagglutination inhibition (HAI) assay is an established technique for assessing influenza immunity, through measurement of antihemagglutinin antibodies. Improved reproducibility of this assay is required to provide meaningful data across different testing laboratories. This study assessed the impact of harmonizing the HAI assay protocol/reagents and using standards on interlaboratory variability. Human pre- and postvaccination sera from individuals (*n* = 30) vaccinated against influenza were tested across six laboratories. We used a design of experiment (DOE) method to evaluate the impact of assay parameters on interlaboratory HAI assay variability. Statistical and mathematical approaches were used for data analysis. We developed a consensus protocol and assessed its performance against in-house HAI testing. We additionally tested the performance of several potential biological standards. In-house testing with four reassortant viruses showed considerable interlaboratory variation (geometric coefficient of variation [GCV] range of 50% to 117%). The age, concentration of turkey red blood cells, incubation duration, and temperature were key assay parameters affecting variability. Use of a consensus protocol with common reagents, including viruses, significantly reduced GCV between laboratories to 22% to 54%. Pooled postvaccination human sera from different vaccination campaigns were effective as biological standards. Our results demonstrate that the harmonization of protocols and critical reagents is effective in reducing interlaboratory variability in HAI assay results and that pools of postvaccination human sera have potential as biological standards that can be used over multiple vaccination campaigns. Moreover, the use of standards together with in-house protocols is as potent as the use of common protocols and reagents in reducing interlaboratory variability.

**IMPORTANCE** The hemagglutination inhibition (HAI) assay is the most commonly used serology assay to detect antibodies from influenza vaccination or influenza virus infection. This assay has been used for decades but requires improved standardization of procedures to provide meaningful data. We designed a large study to assess selected parameters for their contribution to assay variability and developed a standard protocol to promote consistent HAI testing methods across laboratories. The use of this protocol and common reagents resulted in lower levels of variability in results between participating laboratories than achieved using in-house HAI testing. Human sera sourced from vaccination campaigns over several years, and thus including antibody to different influenza vaccine strains, served as effective assay standards. Based on our findings, we recommend the use of a common protocol and/or human serum standards, if available, for testing human sera for the presence of antibodies against seasonal influenza using turkey red blood cells.

## INTRODUCTION

Annual vaccination is currently the most effective method of reducing morbidity and mortality associated with seasonal influenza, a disease that causes an estimated 3 to 5 million serious cases per year and results in 290,000 to 650,000 deaths (1). Seasonal influenza virus strains are recommended for inclusion in a trivalent or quadrivalent vaccine twice yearly, in the northern and southern hemispheres.

The hemagglutination inhibition (HAI) assay is a widely used and long-established technique for assessing influenza immunity, through measurement of antihemagglutinin antibodies. A robust correlate of protection for this assay remains undetermined. An HAI titer of approximately 1:40 was initially described as conferring 50% protection in 1972 (2) and remains a widely used correlate of protection today (3). In order to apply such a correlate effectively and meaningfully, reliable data are needed, using serology assays and reagents that have been standardized across different laboratories.

FLUCOP is a large consortium of 22 members from eight European countries, encompassing academia, vaccine manufacturers, and public health authorities, supported by the Innovative Medicines Initiative Joint Undertaking (IMIJU). The FLUCOP project aims to develop a toolbox of standardized assays to facilitate the development of existing and novel influenza vaccines.

The two main approaches to bioassay standardization are assay harmonization and the use of biological standards. Assay harmonization can be achieved through the use of consensus protocols and central provision or harmonization of critical reagents. However, moving to a consensus protocol may be difficult to implement in laboratories that already use validated protocols for their assays used in registration dossiers; furthermore, the use of common reagents may be limited by availability or cost. In such cases, biological standards can be used to standardize assays and convert readings to normalized values or international units (IU), irrespective of the method used, facilitating comparisons across studies and between laboratories. Previous studies have demonstrated the beneficial effect of using standards on interlaboratory variability of assays measuring influenza immunity (4–8). These studies, however, have typically tested small panels of sera and a limited number of influenza virus strains.

In the current study, we used a data-driven, design of experiment (DOE) method, based on the assimilation of numerous protocols from FLUCOP partners, and a large panel of sera that span the dynamic range of the HAI assay and multiple influenza virus strains, to develop a consensus HAI standard operating protocol (SOP) using turkey red blood cells (TRBCs) (see Text S1 in the supplemental material). In a confirmatory analysis, we evaluated the impact on interlaboratory assay variability of using the consensus SOP, with different potential biological standards, with or without common reagents.

10.1128/mSphere.00567-21.1 TEXT S1 Consensus protocol used for collaborative study (turkey red blood cells). Download Text S1, PDF file, 0.6 MB. Copyright © 2021 Waldock et al. 2021 Waldock et al. https://creativecommons.org/licenses/by/4.0/ This content is distributed under the terms of the Creative Commons Attribution 4.0 International license.

## RESULTS

### Development of a FLUCOP consensus protocol using a design of experiment (DOE) approach. (i) Identification of key assay parameters.

**FIG 1:**
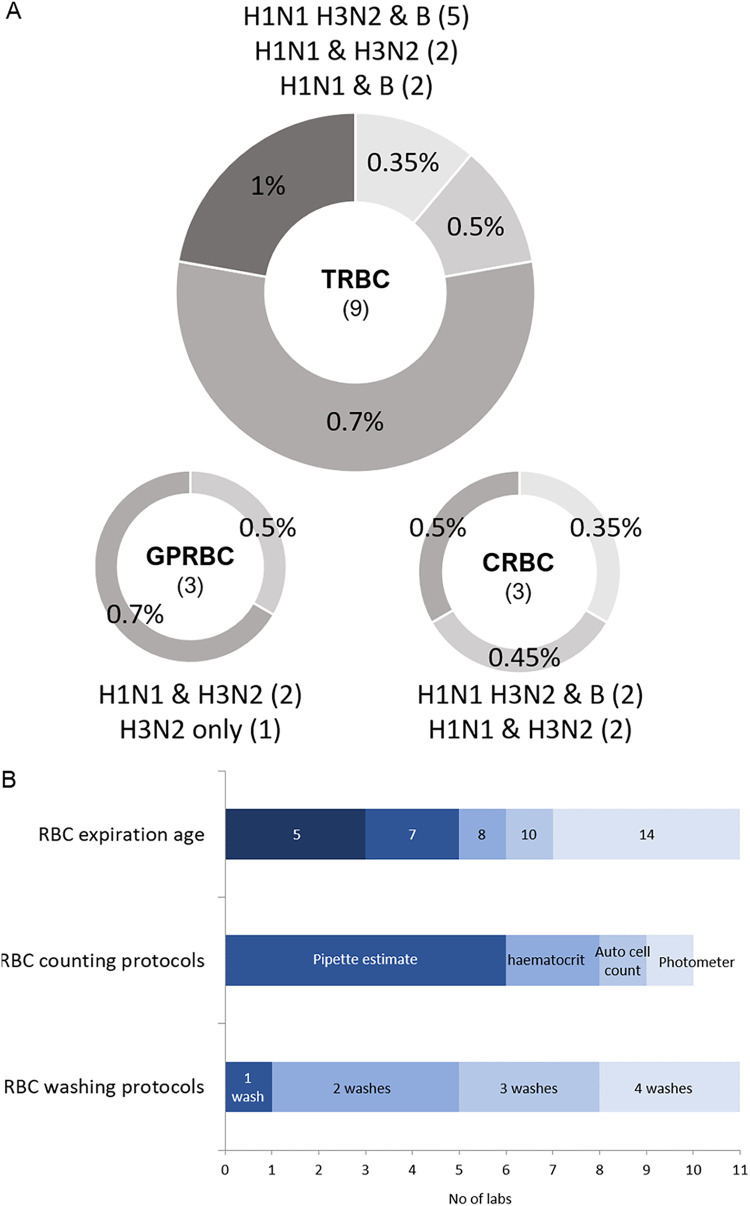
Overview of variation in RBCs used in HAI protocols. (A) Use of RBCs in existing HAI protocols across 11 labs. Turkey RBCs (TRBCs), chicken RBCs (CRBCs), and guinea pig RBCs (GPRBCs) are used by labs for testing different influenza virus strains (above and below pie charts) at different concentrations (% in pie charts). The number of labs is shown in parentheses. (B) Different methods for storing (RBC expiration age in days), counting, and preparing RBCs are currently used across the 11 surveyed labs (only 10 of 11 labs returned data for RBC counting protocols).

To analyze the interlaboratory variability in HAI operating methods, a survey was sent to FLUCOP partners to collect information on their respective HAI protocols. The survey included 62 questions, covering all steps of the protocol, split into seven sections covering (i) red blood cells (RBCs), (ii) serum, (iii) viruses, (iv) hemagglutination (HA), (v) hemagglutination inhibition (HAI), (vi) acceptance criteria, and (vii) controls and standards. Variability was observed in each section of the HAI assay protocol; of 62 survey questions, only 13 (21%) had consistent responses across the laboratories. For 11 (18%) of the questions, variability in the answers was high (≥4 different answers per question) particularly in relation to RBCs (see Fig. S1 in the supplemental material). Some survey forms returned were incomplete, resulting in apparent gaps in the information provided, particularly for sections on “antigen,” “acceptance criteria,” and “controls and standards.” Figure 1 details some of the responses given for use of RBCs, showing the variability in RBC species (and corresponding strains tested with those RBCs) and % concentration (Fig. 1A), as well as storage, washing, and preparation of RBCs (Fig. 1B). There is a clear lack of harmonization across the surveyed labs.

10.1128/mSphere.00567-21.2 FIG S1 Overview of the variation in HAI assay protocols. Data represent responses from 11 FLUCOP partners to a survey on in-house HAI assays. Each bar represents a different question in the survey. The *y* axis represents the number of surveyed labs that gave a response. A different color is added to each bar for each different answer given, so consistent answers have little change in color and variable answers have large changes in color. (1) RBCs; (2) serum; (3) antigen; (4) HA; (5) HAI; (6) acceptance criteria; (7) controls and standards. *Where all laboratories responded and gave the same answer (in this example, all labs used V microtiter plates for HAI assays), the height of the bar is 11, and only 1 color is used. ^Where 10 laboratories responded but provided 8 different answers (in this example, control sera used in the HA assay), the height of the bar is 10 and the bar contains 8 different colors. Download FIG S1, TIF file, 0.4 MB. Copyright © 2021 Waldock et al. 2021 Waldock et al. https://creativecommons.org/licenses/by/4.0/ This content is distributed under the terms of the Creative Commons Attribution 4.0 International license.

Considering the results of the survey, FLUCOP partners agreed on a common HAI protocol for use with TRBCs. In parallel, the following six parameters of the HAI assay were identified as ones likely to have a significant impact on assay variability: (i) turkey RBC (TRBC) concentration, (ii) TRBC age (days from bleeding), (iii) duration for serum/virus incubation, (iv) temperature for serum/virus incubation, (v) duration for serum/virus/TRBC incubation, and (vi) temperature for serum/virus/TRBC incubation.

### (ii) Use of a design of experiment (DOE) to analyze the impact of 6 selected parameters on assay variability and HAI titers.

**FIG 2:**
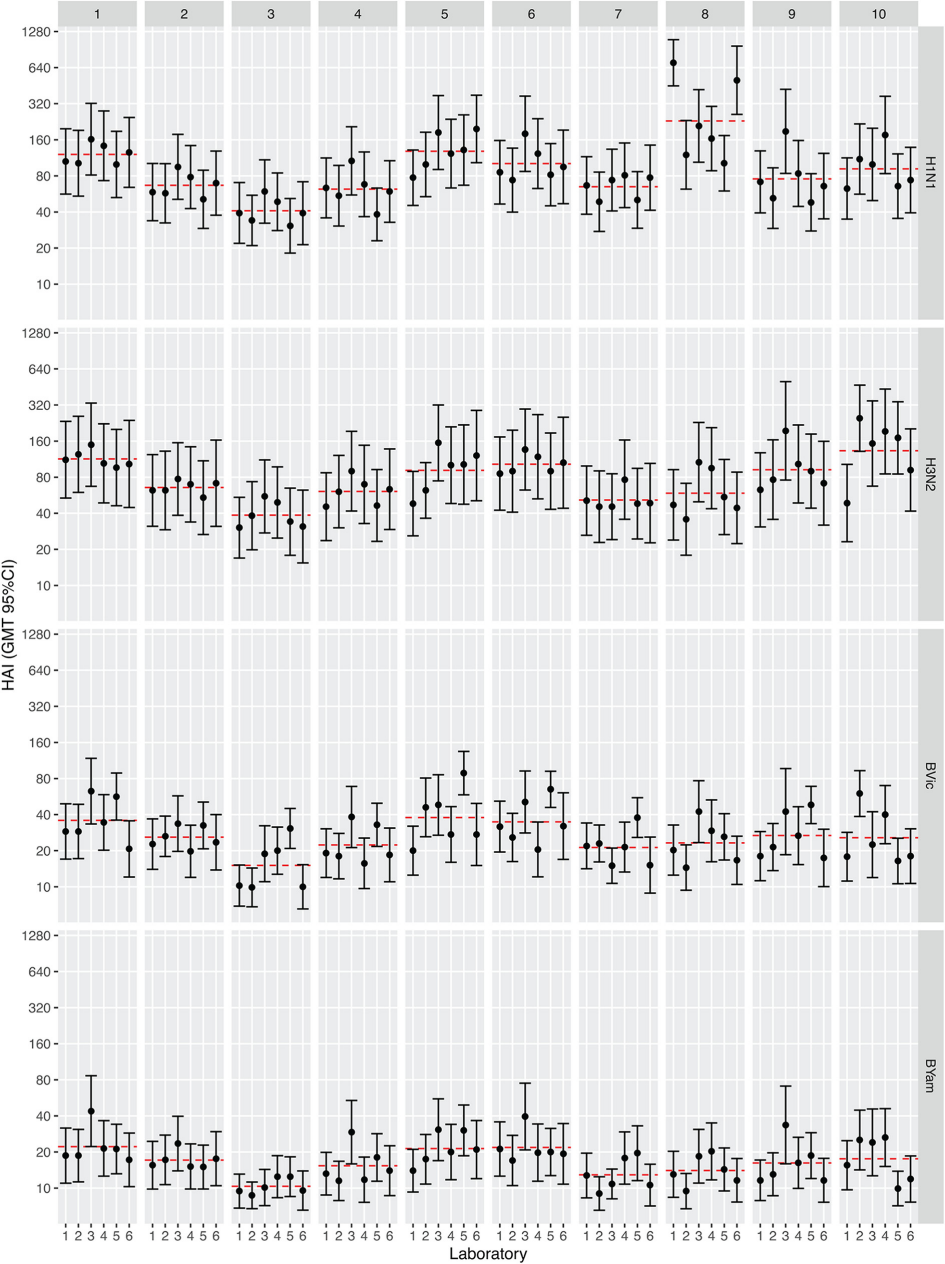
The impact of selected parameters on HAI assay geometric mean titers (GMTs). Ten sets of conditions were tested using a common source of antigen. GMTs of 30 samples (combined replicates) per condition and strain are shown (Table 1 shows design of experiment). Condition 9 is the consensus protocol, and 10 is each laboratory’s in-house protocol. GMTs are depicted with 95% confidence intervals (CI). The red dashed line indicates the GMT per condition across all testing laboratories.

**FIG 3:**
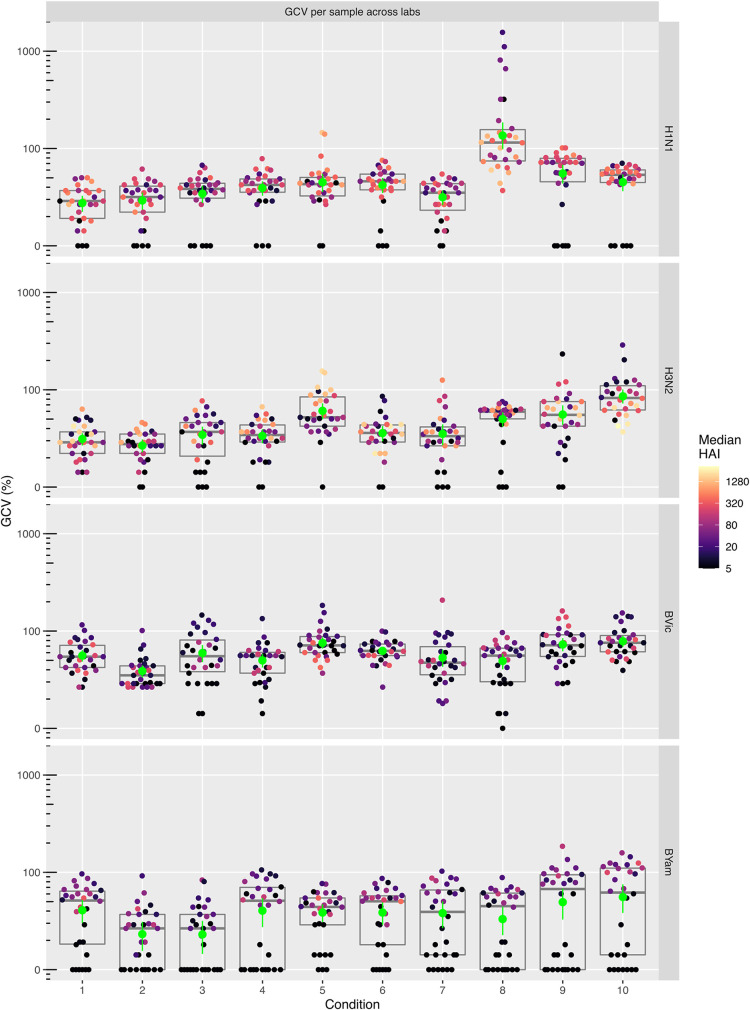
The impact of selected parameters on HAI assay geometric coefficient of variation (%GCV). Ten sets of conditions were tested using a common source of antigen. %GCVs of 30 samples (combined replicates) per condition and strain are shown across all 6 testing laboratories (Table 1 shows design of experiment). Condition 9 is the consensus protocol, and 10 is each laboratory’s in-house protocol. Boxes indicate median and quartile ranges; green points indicate mean %GCV with 95% confidence intervals.

A fractional factorial (2^6-3^) DOE, incorporating high and low settings for each parameter, was used to select a minimal number of sets of experimental run conditions, 1 to 8 (from a potential 2^6^, *n* = 64, combinations) (Table 1). Two additional runs were included for each laboratory: the consensus SOP (run condition 9) and in-house laboratory-specific protocol (10). Each laboratory conducted 20 independent experiments (10 sets of run conditions in duplicate) on a panel of 30 samples (serum panel 1 [see Materials and Methods]). Common stocks of critical reagents were used across the laboratories, including four egg-grown reassortant viral strains: A/California/07/2009 NYMC X-181 (H1N1), A/Switzerland/9715293/2013 NIB-88 (H3N2), B/Brisbane/60/2008 NYMC BX-35 (B/Victoria lineage), and B/California/12/2015 NYMC BX-59A (B/Yamagata lineage). A total of 19,200 assay results were generated (4,800 HAI titers for each influenza virus strain). Geometric mean titers (GMTs) calculated for each set of run conditions tested, per seasonal influenza virus, showed a high level of variability between laboratories for some run conditions, in particular condition 8 for H1N1, with GMTs ranging from 102 to 702, while other sets of conditions gave relatively consistent results across laboratories, in particular condition 2 with GMTs ranging from 51 to 95 (Fig. 2). Overall, the interlab variability, measured by geometric coefficients of variation (%GCV), ranged from 45% to 86%, depending on the strain, when using an in-house protocol together with common reagents. A number of conditions showed consistent reduction in %GCV compared to in-house testing of common antigens across the four virus strains, namely, conditions 1, 2, and 7 (Fig. 3).

**TABLE 1:** Design of experiment—2^6-3^ fractional factorial design^*a*^

Expt condition	TRBC		Incubation 1		Incubation 2	
	Age (days from bleeding)	Concn (%)	Time, min (virus + serum)	Temp (°C)	Time, min (virus + serum + TRBCs)	Temp (°C)
1	1 to 4	0.35	90	37	30	RT
2	7 to 11	0.35	30	RT^*c*^	30	37
3	1 to 4	1.00	30	RT	60	RT
4	7 to 11	1.00	30	37	30	RT
5	1 to 4	0.35	30	37	60 min	37
6	7 to 11	0.35	90	RT	60 min	RT
7	1 to 4	1.00	90	RT	30 min	37
8	7 to 11^*b*^	1.00	90	37	60 min	37
9	1 to 4	0.50	60	RT	45 min	RT
10						
Lab 1	1 to 5	0.70	60	RT	30 min	RT
Lab 2	1 to 7	0.50	60	RT	30 min	RT
Lab 3	1 to 15	0.35	60	RT	60 min	RT
Lab 4	1 to 4	0.50	60	37	45 min	37
Lab 5	1 to 7	0.45	60	RT	60 min	RT
Lab 6	1 to 5	0.70	60	RT	30 min	RT

a Design of experiment for assessing selected assay parameters. Each laboratory tested panel 1 under the following 10 sets of conditions, in duplicate. Experimental condition 9 is the consensus SOP, and 10 is the in-house SOP.

b The fractional factorial design required TRBCs aged 7 to 11 days in condition 8; however, by mistake, all labs were instructed to use TRBCs age 1 to 4 days. A statistical simulation was conducted and showed the error had minimal to no impact on the outcomes and conclusions of the statistical model.

c RT, room temperature.

**FIG 4:**
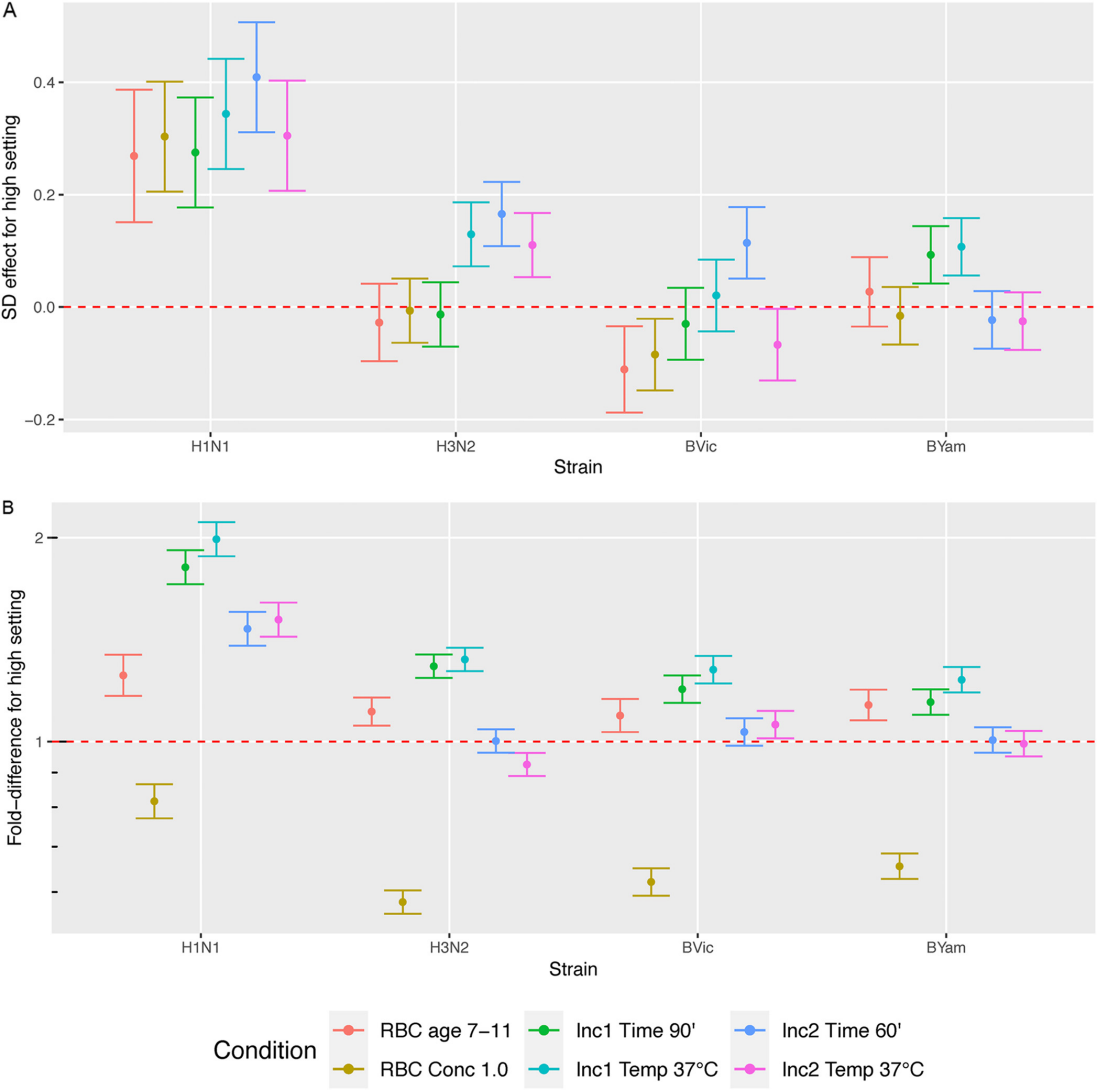
The effect of the parameters tested on variation between laboratories (difference in SD of log_2_ HAI = difference in wells added due to high setting) (A) and fold change in overall GMT (B). The fold difference using the “high setting” of each tested parameter compared to the “low setting” is plotted for each virus strain (see Table S1). TRBC, turkey red blood cells; Inc1, incubation step involving virus and serum sample; Inc2, incubation step involving virus, serum, and TRBCs.

In a statistical analysis, the impact of using the high setting compared to the low setting for each parameter (see Table S1 for high, low, and normal parameter settings) on GMTs and standard deviation (SD) between participating laboratories was evaluated (Fig. 4). Parameter settings that reduced variation, with minimal effect on GMTs, were earmarked as the most suitable to be used in a common protocol. For example, the high setting for TRBC age (using older [7 to 11 days] TRBCs in the HAI assay) had a mixed impact on SD, and it increased GMTs. Therefore, the “low” setting (fresh TRBCs of 1 to 5 days) was deemed to be the optimal setting.

10.1128/mSphere.00567-21.7 TABLE S1 Parameter settings, for analysis in a fractional factorial DOE. Download Table S1, DOCX file, 0.01 MB. Copyright © 2021 Waldock et al. 2021 Waldock et al. https://creativecommons.org/licenses/by/4.0/ This content is distributed under the terms of the Creative Commons Attribution 4.0 International license.

**FIG 5:**
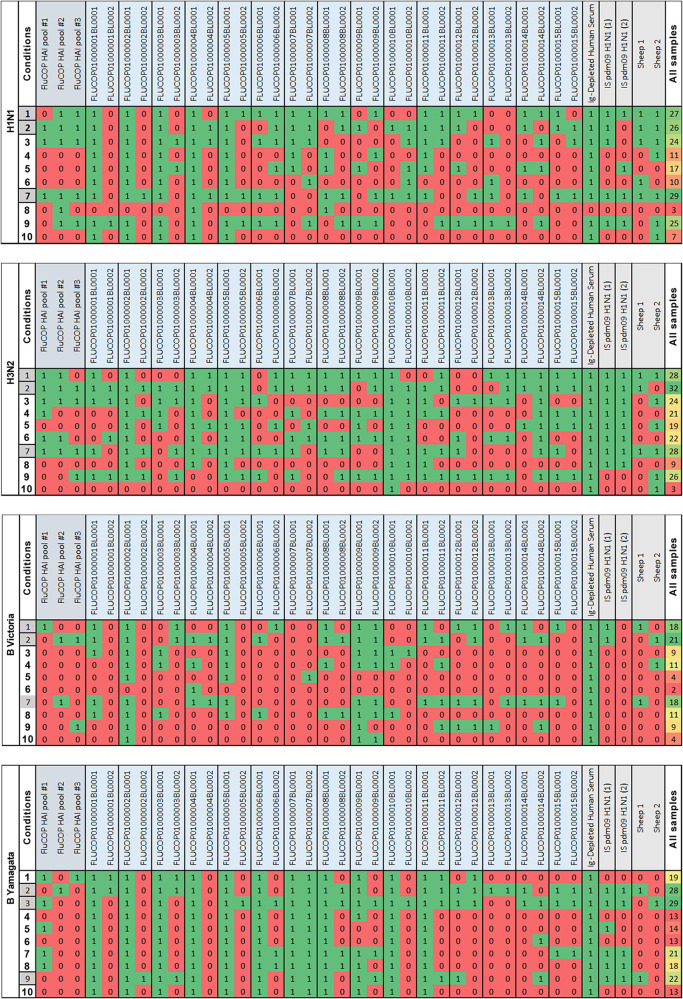
Mathematical analysis of interlaboratory variation across 10 sets of conditions tested. Each sample was scored for high (red or 0) or low (green or 1) variability following a mathematical approach to analyzing the data. The total score across all samples is shaded from high-med-low as green-yellow-red (All samples). The best three sets of conditions are highlighted in gray in the “Conditions” column. Table 1 details the parameters used in conditions 1 to 10.

A mathematical analysis approach to determine the association of the 10 different sets of conditions with high or low levels of interlab variation (Fig. 5) showed almost identical findings as the statistical approach. Using the Q-finder algorithm, we confirmed the results identified by the statistical approach and did not identify any other conditions that would be predicted to give less-variable results than those already tested. The two methods identified the same sets of conditions that give the lowest source of interlaboratory variation for each virus tested (namely, conditions 1, 2, and 7 with some strain-specific differences [Fig. 5]).

From these analyses we selected the most common parameter settings found under low-variation conditions, avoided parameter settings that adversely affect GMTs, and took into account practicalities such as the ease of use and availability of reagents, and generated a consensus FLUCOP SOP for HAI using TRBCs for testing in further studies (see supplementary material at https://doi.org/10.6084/m9.figshare.14822475). The optimal settings identified for the parameters were as follows: fresh TRBCs (1 to 5 days), 0.5% TRBC concentration, 90-min incubation period 1 (virus and serum), room temperature for incubation 1; 30-min incubation period 2 (virus, serum, and TRBC), room temperature for incubation 2.

### Confirmatory study: the impact of protocol harmonization, common source reagents, and biological standards on HAI assay variability.

To verify that the DOE/mathematical analysis approaches had identified optimal conditions, we performed a confirmatory study in which we tested the effect of using the consensus SOP against in-house testing, in combination with in-house or commonly sourced reagents, and assessed the effect of use of different biological standards on interlaboratory assay variability.

### (i) Harmonization of protocol and reagents.

**FIG 6:**
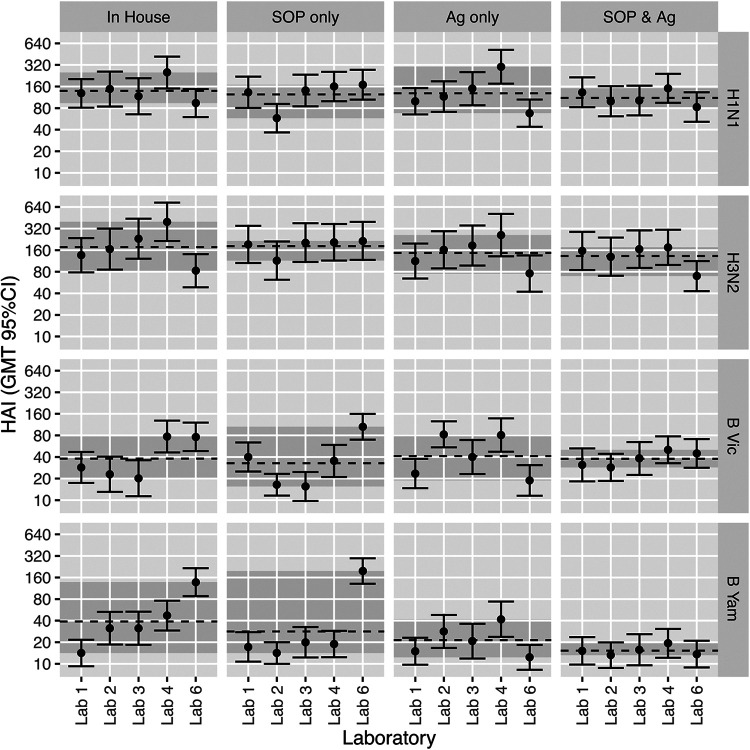
The impact of using a common SOP and/or a common source antigen (Ag) on HAI titer GMTs. GMTs of 30 samples (combined replicates) per condition and strain are shown. Titers plotted are raw calculated titers. GMTs are depicted with 95% confidence intervals. The black dashed line indicates the GMT per condition across all testing laboratories. The gray-shaded area shows the range of GMTs across all testing laboratories. Conditions are as follows: in-house, in-house assay protocol; SOP, FLUCOP consensus protocol; Ag, common source antigen/virus.

Four experimental conditions were tested using a 2^2^ factorial DOE: (i) in-house protocol and in-house virus/reagents, (ii) consensus SOP and in-house virus/reagents, (iii) in-house protocol and common source virus/reagents, and (iv) consensus SOP and common source of virus/reagents. Each of these run conditions was tested in duplicate using serum panel 2 (*n* = 30) and four controls, using the same four influenza virus strains used in the initial DOE, to generate 5,440 HAI titers across five testing laboratories. For all strains, the most homogeneous GMT distribution across laboratories was observed when both consensus SOP and common source of virus were used, especially for B strains (Fig. 6). Using a consensus SOP and common source antigen reduced the GMT range for each strain compared to in-house testing: for H1N1 from 94–251 to 83–151, for H3N2 from 82–398 to 70–175, for B Victoria from 20–77 to 29–50, and for B Yamagata from 14–137 to 14–19 **(**Fig. 6).

**FIG 7:**
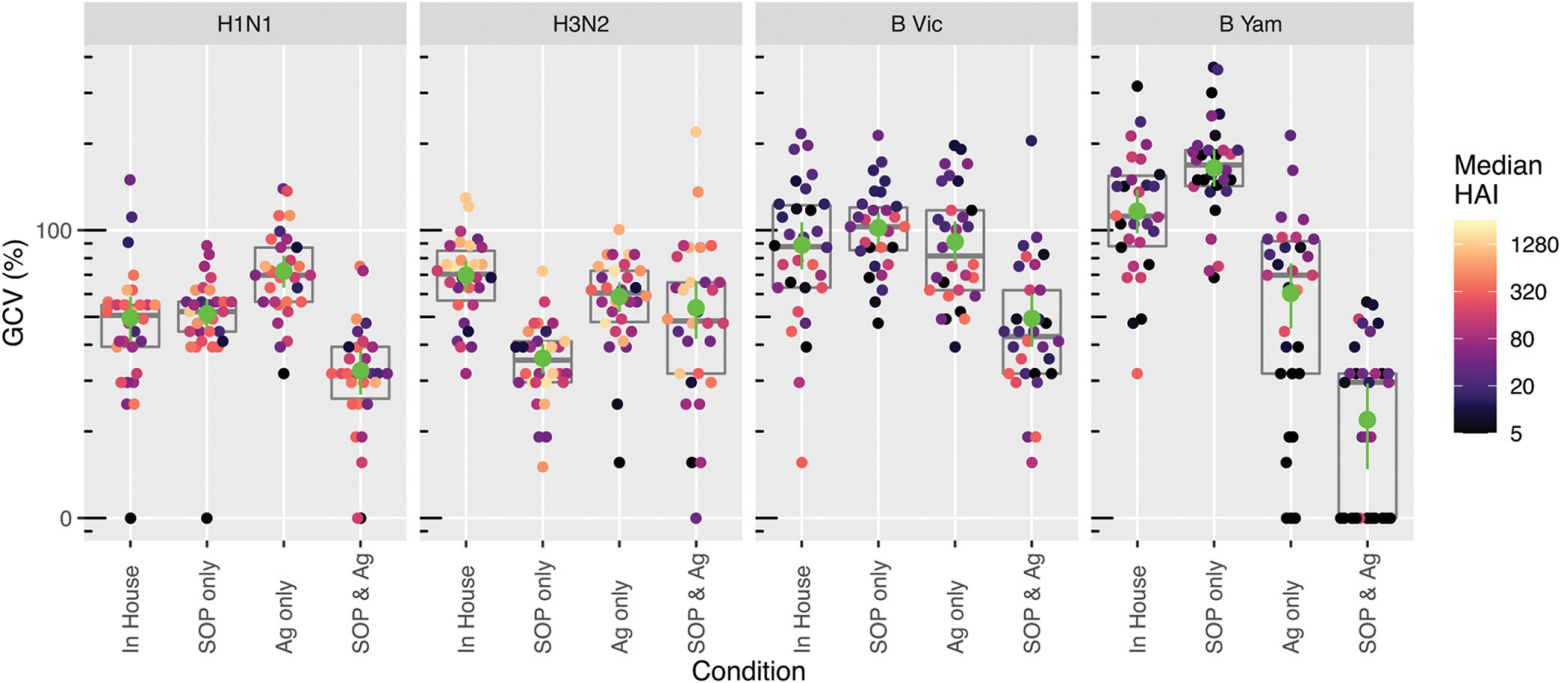
The impact of using a consensus SOP and/or a common source of antigen on HAI titer geometric coefficient of variation (%GCV). %GCVs of 30 samples per condition (replicates combined) across all testing laboratories are shown for four seasonal influenza viruses. Plots show raw calculated GCVs. Boxes indicate median and quartile ranges; green points indicate mean %GCV with 95% confidence intervals. Median HAI titers of individual samples are shown by a color gradient.

The use of the consensus SOP with a common source virus significantly reduced interlaboratory variation compared to the use of in-house protocols for H1N1 (%GCV reduced from 50% to 32% [*P* = 0.0052]), H3N2 (%GCV reduced from 70% to 54% [*P* = 0.0228]), B Victoria (%GCV reduced from 89% to 49% [*P* = 0.0047]), and B Yamagata (%GCV reduced from 117% to 22% [*P* < 0.00001]) strains (Fig. 7). For both B strains, the use of common stock virus alone reduced the variability, although this was significant only for the B Yamagata strain (*P* = 0.000014). This is likely due to the mixed use of ether-split and native B antigens in different labs’ in-house testing, as ether splitting of antigen is known to increase HAI titers. For the H3N2 strain, the best condition tested was the use of the FLUCOP consensus SOP with in-house antigen (Fig. 7). However, high levels of intralab variation were observed for one laboratory when testing the consensus SOP and common source antigen, increasing the %GCV for this condition. For the remaining 4 laboratories, the consensus SOP and common source antigen give the lowest variability.

In a limited small-scale study, we expanded our testing to multiple influenza virus strains and nonavian RBCs. First, we tested wild-type (WT) egg- and cell-grown viruses with matching antigenicity to the H1N1, H3N2, and B Victoria reassortants in 2 laboratories. In-house protocols or the consensus SOP and common source antigen were used to test a 20-sample serum panel (panel 3). The consensus SOP with common source virus gave significantly lower %GCV for all WT egg-grown viruses and 2/3 WT cell-grown viruses (Fig. S2). Second, considering the increasing number of H3N2 strains over the past decade that no longer agglutinate avian RBCs and demonstrate agglutination via NA activity (9), we also tested a WT cell-grown H3N2 virus with and without the NA inhibitor oseltamivir. A 15-sample panel (panel 4) was tested using in-house protocols and a harmonized SOP for guinea pig RBCs (GPRBCs) with common source antigen in two laboratories. The use of oseltamivir appears to introduce interlaboratory variation for in-house testing (Fig. S3). The consensus SOP and common source virus reduces %GCV in the presence of oseltamivir, highlighting the importance of further studies and harmonization of testing for GPRBCs and when using NA inhibitors. These small studies further support the use of harmonized protocols but warrant further investigation for robust statistical analysis.

10.1128/mSphere.00567-21.3 FIG S2 Comparison of WT egg- and cell-grown viruses tested in HAI under in-house or FLUCOP (SOP and Ag) conditions. A 20-sample serum panel was tested with WT viruses A/California/07/2009 (Egg) and A/California/04/2009 (Cell) (H1N1), A/Switzerland/9715293/2013 Egg and Cell (H3N2), and B/Brisbane/60/2008 (Egg) and B/Texas/02/2013 (Cell) (B Vic). Viruses were egg isolated and passaged in eggs (Egg) or cell isolated and passaged in cells (Cell). Two laboratories tested the panel using their in-house protocols (In-house) or using the FLUCOP SOP and common source antigen (SOP and Ag). %GCV/sample is plotted, colored by median HAI titer. Using the FLUCOP SOP and Ag gives significantly lower %GCV for H1N1 Egg antigen (*P* = 0.0000284), H3N2 Egg antigen (*P* = 0.0007062), H3N2 Cell antigen (*P* = 0.0000425), B Vic Egg antigen (*P* = 0.00028), and B Vic Cell antigen (*P* = 0.0000193). Download FIG S2, TIF file, 0.1 MB. Copyright © 2021 Waldock et al. 2021 Waldock et al. https://creativecommons.org/licenses/by/4.0/ This content is distributed under the terms of the Creative Commons Attribution 4.0 International license.

10.1128/mSphere.00567-21.4 FIG S3 Comparison of WT cell H3N2 virus tested in GPRBCs under in-house or FLUCOP (SOP and Ag) conditions. (A) %GCV plots for A/Switzerland/9715293/2013 in-house versus FLUCOP SOP/antigen with or without oseltamivir. The %GCV per sample tested in two laboratories in duplicate is plotted. Samples are colored by median HAI titer. Without oseltamivir (left), both in-house testing and FLUCOP testing (SOP and Ag) show consistent %GCV per sample. With oseltamivir (right), FLUCOP testing (SOP and Ag) results in significantly lower %GCV per sample than in-house testing across the two testing laboratories (*P* = 0.000153). (B) Overall GMT across the 15-sample serum panel is shown for testing without (left two columns) and with (right two columns) oseltamivir. Download FIG S3, TIF file, 0.1 MB. Copyright © 2021 Waldock et al. 2021 Waldock et al. https://creativecommons.org/licenses/by/4.0/ This content is distributed under the terms of the Creative Commons Attribution 4.0 International license.

### (ii) Biological standards.

**FIG 8:**
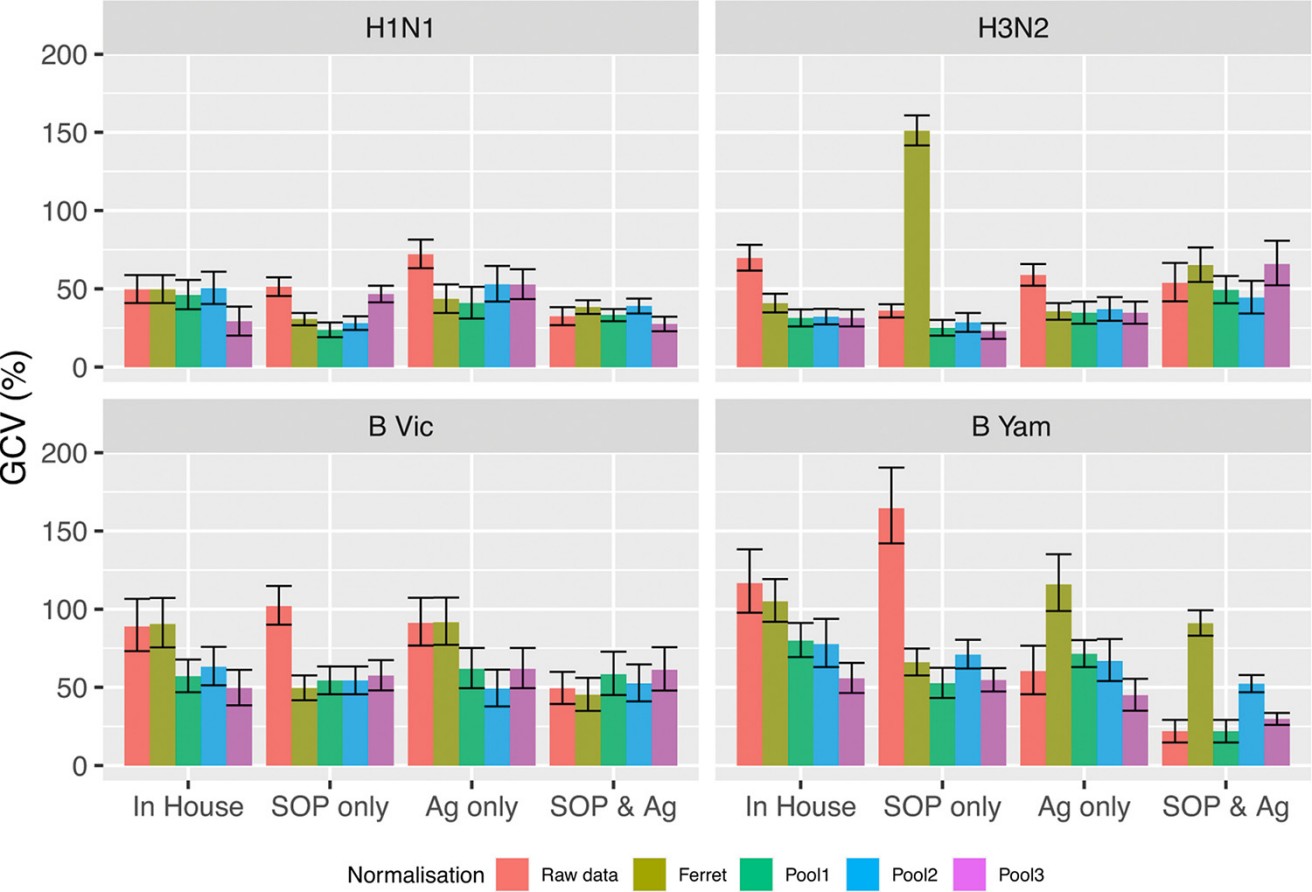
Performance of biological standards. Interlaboratory variation across all testing laboratories was calculated as the mean standard deviation for the log_2_ HAI titer (of the combined replicates) per sample and condition across the labs. The resulting summary statistics were then converted to %GCV. %GCV is plotted for the following: in-house testing (red) and in-house testing when HAI titers are expressed relative to a homologous ferret serum (olive) and when HAI titers are expressed relative to the three pools of postvaccination human sera (2010–2011 season, green; 2012–2013 season, blue; 2015–2016 season, pink) (composition of the vaccines is given in Table 2).

Three pools of postvaccination human sera corresponding to three different vaccination campaigns between 2010 and 2015 (Table 2) were tested alongside ferret and sheep sera as potential biological standards. The use of human and ferret sera as standards for in-house HAI testing reduced interlaboratory variation compared to in-house testing alone, albeit to a limited extent using ferret sera (Fig. 8). Interlaboratory variability was not reduced when using sheep sera as a standard (data not shown). The pooled human sera from the 2015–2016 vaccination campaign (in which individuals were vaccinated with strains matched to those used in HAI testing) performed the best, with GCVs reduced from 50% to 117% (no normalization) to 29% to 56% across the four virus strains (Fig. 8). The performance of the antibody standards deteriorated with pooled sera from earlier, mismatched vaccination campaigns, with GCVs of 32% to 78% for the 2012/2013 standard and 31% to 80% for the 2010/2011 standard. Notably, a reduction in GCV was observed for all three pools of human sera, regardless of year of vaccine campaign, demonstrating the potential for this type of material as a biological standard that could be used over multiple years.

**TABLE 2:** Three pools of postvaccination human sera included as potential standards

Pool	Vaccine campaign yr	H1N1	H3N2	B Victoria	B Yamagata
Pool 1 (*n* = 9 subjects)	2010–2011	A/California/07/2009	A/Victoria/210/2009	B/Brisbane/60/2008	NA^*a*^
Pool 2 (*n* = 9 subjects)	2012–2013	A/California/07/2009	A/Victoria/361/2011	NA	B/Texas/06/2011
Pool 3 (*n* = 9 subjects)	2015–2016	A/California/07/2009	A/Switzerland/9715293/2013	NA	B/Phuket/3073/2013

a NA indicates where B strains were not present in the recommended vaccine composition for trivalent vaccines.

### (iii) Harmonization versus biological standards.

To assess which approach to standardization gave the best results, we compared the improvement in interlaboratory variability achieved through the use of a harmonized protocol and common stocks of critical reagents, with the use of a standard alone, or a combined approach. For H1N1, H3N2, and B Victoria, the use of pooled human sera from individuals vaccinated with the same influenza virus strain as that used in the assay, using in-house testing, consistently gave the lowest %GCV across participating laboratories. For B Yamagata, strict harmonization using a consensus SOP and common antigen gave the lowest %GCV across testing laboratories; however, normalization with human serum standards also reduced interlaboratory variation (Fig. 8). Figures S2 and S3 show the impact of harmonization and using a human serum standard on GMT (Fig. S4) and %GCV (Fig. S5) for each condition and strain tested in the study.

10.1128/mSphere.00567-21.5 FIG S4 The impact of using a common SOP and/or a common source antigen on HAI titer GMTs. GMTs of 30 samples (combined replicates) per condition and strain are shown. Titers plotted are raw reported titers (orange) or have been normalized using human serum pool 1 (green, 2010–2011 season), pool 2 (blue, 2012–2013 season), or pool 3 (pink, 2015–2016 season). GMTs are depicted with 95% confidence intervals. The black dashed line indicates the GMT per condition across all 6 testing laboratories. Download FIG S4, TIF file, 0.1 MB. Copyright © 2021 Waldock et al. 2021 Waldock et al. https://creativecommons.org/licenses/by/4.0/ This content is distributed under the terms of the Creative Commons Attribution 4.0 International license.

10.1128/mSphere.00567-21.6 FIG S5 The impact of using a consensus SOP and/or a common source of antigen on HAI titer geometric coefficient of variation (%GCV). %GCVs of 30 samples per condition (replicates combined) across all testing laboratories are shown. Column 1 plots raw reported titers. Columns 2 to 4 show titers after normalization using human serum pools1 to 3, respectively. Boxes indicate median and quartile ranges; green points indicate mean GCV with 95% confidence intervals. Download FIG S5, TIF file, 0.4 MB. Copyright © 2021 Waldock et al. 2021 Waldock et al. https://creativecommons.org/licenses/by/4.0/ This content is distributed under the terms of the Creative Commons Attribution 4.0 International license.

Overall, the use of a consensus SOP and common reagents combined reduced the %GCV compared to the use of in-house procedures and reagents across all influenza virus strains tested. Using a matched human serum standard with in-house testing has the greatest impact on reducing %GCV overall as %GCVs were lowest under these conditions for three out of four strains tested. Interestingly, a combination of harmonization and using a biological standard did not offer an advantage over using a standard alone for in-house testing or using strict harmonization (Fig. 8).

## DISCUSSION

In our study, in-house HAI testing of four reassortant viruses showed considerable variability in results between laboratories, with GCVs for the different strains of 50% to 117%. The highest levels of variation were for the B influenza virus strains (89% for B Victoria; 117% for B Yamagata). This may be partly explained by the combination of data from assays using native and ether-split B antigens: the common stock virus was native, while some laboratories used ether-split antigens for their in-house assay. Splitting of B viruses substantially increases HAI titers and results in large differences in titers generated compared with native antigen (10).

Previous collaborative studies investigating the standardization of HAI assays showed even greater levels of interlaboratory variation (4, 6–8, 11), with GCVs for HAI titers of 95% to 345% for an H1N1 strain (8), 138% to 261% for H3N2 strains (11), and 183% for a clade 1 H5 virus (4). A 2016 study assessing HAI across a network of laboratories in Canada showed substantial improvement in GCV under strictly standardized conditions (median GCVs of 17% [H1N1] and 39% [H3N2]) (12).

In the current study, the comparison of 11 HAI protocols from FLUCOP consortium members highlighted numerous differences between in-house HAI assay protocols, particularly in relation to RBCs. Previous studies have demonstrated the value of assay harmonization (12) or the use of biological standards to reduce interlaboratory variability (4, 8, 11). We incorporated both approaches to develop a data-driven consensus HAI protocol and then evaluated the impact of protocol harmonization versus use of biological standards on interlaboratory variation in HAI testing. A comparison of results generated from in-house testing with those generated under different standardized conditions suggested that a strict level of assay harmonization is needed to effectively reduce variability across the four virus strains tested in this study. Consistent with previous findings (12), testing under harmonized conditions, including using a common source of antigen and common reagents and following a consensus protocol, significantly reduced GCV to 22% to 54%. This improvement in agreement after harmonization was also observed for small-scale testing of a further 3 WT egg-grown viruses and 2 WT cell-grown viruses and for testing a WT cell H3N2 virus with oseltamivir in GPRBCs, further supporting the use of harmonized protocols and reagents for HAI.

The practicality of introducing such harmonized procedures and reagents across industry, regulatory, and academic laboratories is doubtful, in particular where laboratories are required to validate their HAI assay. In such cases, it may not be possible to adopt a consensus SOP; additionally, the capacity for all laboratories to use the same critical reagents may be limited by reagent availability and cost. Further investigations into how these approaches could be implemented in the real world are required. However, given the improvement observed in interlaboratory variation using the FLUCOP consensus protocol, in particular for H3N2 viruses, we recommend that this protocol be adopted by laboratories where possible. To facilitate the use of the consensus protocol, we developed a training video (see supplementary material at https://doi.org/10.6084/m9.figshare.14822475).

This is the first study to compare the impact of assay harmonization with the use of a standard in interlaboratory variability head-to-head. We tested various materials as potential standards including human, sheep, and ferret sera. When identifying material that could be used as a general antibody standard, considerations such as the volume required for testing, the stability of the material, and the cost of generating and replacing the material need to be taken into account. Other considerations specific to seasonal influenza virus standards should include the immunological history of exposure to influenza of an individual donor/pool of donors, cross-reactivity of virus subtypes or drifted strains within the same subtype, the longevity of the standard as viruses drift from year to year, and the practicalities of defining the units of the standard for multiple influenza strains. Sheep sera, as used in the current study, provide a large volume of antibody standard and have a controlled immunological history (specific to the virus hemagglutinin to which they were hyperimmunized). However, sheep serum standards do not perform consistently well, as shown previously (4) and confirmed in the current study (data not shown). It remains unclear why HAI titers of sheep sera vary widely between laboratories.

While the advantage of ferret sera is their specificity, which can be tailored to match any influenza virus strain used in vaccine studies, the main disadvantage when considering them for use in international influenza virus antibody standards is the small volume of serum that can be obtained from individual animals; the necessity to inoculate several ferrets to create suitable volumes represents an ethical burden due to the animal work required. In this study, we showed that ferret sera reduced interlaboratory variability, but to a lesser extent than strict protocol harmonization, or the use of pooled human sera as a standard. Thus, ferret sera may not be appropriate as antibody standards for seasonal influenza viruses.

The greatest reduction in interlaboratory variation for three out of four influenza virus strains tested in this study was achieved using pooled postvaccination human sera as a biological standard. We tested three pools of sera spanning 5 years of influenza vaccine campaigns to address the issue of drift in seasonal influenza viruses. The antibody standards from sera of volunteers exposed to virus strains that most closely matched the virus strains tested showed the greatest reduction in %GCV. However, all of the three pooled sera tested reduced %GCV for each virus strain tested. This suggests that human serum standards made from post-quadrivalent-vaccination pools can function well over multiple influenza seasons, which is an important factor in the life span of a serology standard.

The performance of the three pooled sera as standards for the H1N1 virus differed, despite containing postvaccination sera from individuals vaccinated with the same H1N1 virus, A/California/07/2009. One possible explanation is the complex immunological history of the different donors for each of the three pools, with each pool having unique antibody profiles that may differ in terms of previous exposure to natural influenza virus infection or previous vaccination (13–15). The impact of these differences between pooled human sera may be overcome by using larger numbers of individuals in each pool.

In the comparison of the impact of harmonization and use of biological standards separately, or as a combined approach, the most consistent reduction in interlaboratory variation was achieved with in-house testing with a pooled human serum standard (range of %GCV across the four virus strains reduced from 50% to 117% to 29% to 56% with a matching human serum standard). We did not see a cumulative effect in reduction of interlaboratory variability for the combined use of harmonization and biological standards (GCV, 27% to 66% across the four strains). It may be that we are close to the limits of improvement in agreement using both approaches, and remaining variability is inherent to the existing assay. For example, it is not possible to use a common source of RBCs in multiple laboratories, and without a standardized alternative, such as the development of “artificial” RBCs or beads coated in sialic acid for use in HAI, the variability introduced by RBCs cannot be reduced. As both approaches to standardization give good results, we encourage the adoption of consensus SOPs in the absence of commercially available seasonal influenza virus standards and highlight the importance of developing such standards for seasonal influenza virus serology.

### Limitations of the study.

This study has primarily focused on the development and testing of an HAI protocol for use with TRBCs. In the past decade there has been an increase in H3N2 influenza viruses that fail to agglutinate avian RBCs and thus cannot be assessed in HAI using TRBCs. In addition to this, many H3N2 virus strains have been shown to agglutinate via their neuraminidase (9), obscuring the interaction between anti-HA antibodies, virus, and RBCs. An alternative for such virus strains is to test in HAI using guinea pig RBCs (GPRBCs) using a neuraminidase inhibitor such as oseltamivir to prevent NA-mediated agglutination. Using the same data-driven approach to develop a harmonized GPRBC SOP was unfortunately outside the scope of this study. The results from our limited testing indicate that the use of oseltamivir in particular introduces interlaboratory variation, which can be reduced through protocol harmonization and the use of common source reagents. This warrants further investigation, and the evidence-based development of a consensus SOP for GPRBCs and statistically robust testing of that SOP is a high priority for future work.

### Conclusions.

We showed that harmonization through the use of the consensus FLUCOP protocol and common critical reagents significantly reduces interlaboratory variation. We additionally demonstrated that pooled postvaccination human sera can be used as serum standards for at least 5 years following the respective vaccination campaign, provided that matching virus subtypes were included in the vaccine. Moreover, the use of standards together with in-house protocols is as potent as the use of common protocols and reagents in reducing interlaboratory variability. In the absence of existing biological standards for seasonal influenza, we encourage the use of the FLUCOP SOP for HAI testing using TRBCs; further assessment of potential biological standards for use in seasonal influenza serology is ongoing.

## MATERIALS AND METHODS

### Collection of protocols and selection of key assay parameters.

An Excel spreadsheet-based template requesting information on in-house HAI procedures was generated and circulated to 12 FLUCOP partners. Partners were asked to fill in as much information as possible and return this for collation. Each response was added into a database stored at the lead laboratory (National Institute for Biological Standards and Control [NIBSC]). Data were analyzed to evaluate variability at each step of the HAI protocol: RBCs, serum, antigen, hemagglutination (HA), HAI, acceptance criteria, and controls and standards.

FLUCOP partners were also asked to identify six key parameters of the protocol expected to have a major impact on assay variation for further evaluation.

### Participating laboratories for HAI data analysis.

Six laboratories participated in the analyses of the impact of key parameters on HAI data: NIBSC, Public Health England (PHE), Sanofi Pasteur (SP), GlaxoSmithKline (GSK), University of Siena (UNISI), and University of Bergen (UIB).

### Analysis of HAI assay run conditions (factorial DOE).

To establish a manageable experimental design for evaluation of the 6 assay parameters in the initial DOE analysis, we used a fractional 2^6-3^ factorial design. We selected 8 sets of conditions from a total of 2^6^ (*n* = 64) possible combinations of the 6 parameters (Table 1). Two additional sets of run conditions were included in the DOE: testing laboratories’ in-house protocol and a common SOP. Each laboratory conducted 20 independent assay runs to assess 10 sets of run conditions, in duplicate.

In a confirmatory DOE, a 2^2^ factorial design was used to analyze the impact on assay variability of 4 different combinations of consensus versus in-house protocols and common versus in-house reagents. Analysts were blinded to the sample IDs by randomization of the sample location on the plate, including the sample duplicates. The order of assay runs for all viruses was randomized for each laboratory.

### In-house testing.

Participating laboratories carried out HAI testing using their in-house methods. In-house procedures were also used for serum preparation, receptor-destroying enzyme (RDE) treatment, and testing for nonspecific agglutination in serum samples.

### FLUCOP testing.

Each laboratory received a comprehensive workbook on “FLUCOP HAI” testing conditions, specific for each laboratory. This described the order of testing for the 20 assay runs (based on a laboratory-specific randomization plan) and a detailed SOP for the HAI assay (including work instruction templates and data reporting templates). The test conditions were recorded for each assay run according to a laboratory-specific experimental design. Results were collected in an electronic results sheet that formed part of the workbook. Data entry was checked against paper copies for each HAI run prior to submission to minimize data entry errors. The submitted data were checked independently by two laboratories (Quinten and Biomedical Primate Research Centre [BPRC]).

For FLUCOP testing, briefly, serum samples were treated with RDE (Denka Seiken) (4 parts RDE:1 part serum). Diluted sera were treated with packed TRBCs to remove nonspecific agglutinins and transferred to a 96-well plate for testing. Four virus strains were tested in each HAI run. The HA titer of each viral antigen was calculated using a specified concentration of TRBCs and diluted to 4 HA units/25 μl. The HA titer was confirmed using a back titration and adjustments made for dilution as required. Serum samples were titrated across a V-bottomed plate from 1:10 to 1:2,560 in columns 1 to 11. Column 12 served as a serum-only control. Diluted viral antigen was added to the sera (columns 1 to 11) and incubated according to the conditions specified. TRBCs of specified age and concentration were added to all wells and incubated accordingly. The HAI titer was defined as the reciprocal of the last well in which complete inhibition of agglutination was observed. For HAI using guinea pig RBCs (GPRBCs), the same protocol was used with the following modifications: sera were not RBC adsorbed prior to testing (due to limited volumes of GPRBCs), 0.7% GPRBCs in phosphate-buffered saline (PBS)–0.1% bovine serum albumin (BSA) was used for HA/HAI; 25 μl of virus was titrated in 25 μl PBS-0.1% BSA with a final concentration of 20 nM oseltamivir/well for HA determination; sera were titrated in PBS-0.1% BSA with a final concentration (after addition of antigen) of 20 nM oseltamivir/well; incubation time for virus plus sera was 60 min; incubation time for virus plus GPRBCs was 60 min.

### Viruses.

Four strains of the seasonal influenza virus vaccine were used for HAI testing: A/California/7/2009 NYMC X-181 (H1N1), A/Switzerland/9715293/2013 NIB-88 (H3N2), B/Brisbane/60/2008 NYMC BX-35 (B/Victoria lineage), and B/California/12/2015 NYMC BX-59A (B/Yamagata lineage). WT egg- and cell-propagated antigens antigenically similar to the four reassortants were additionally tested. Antigens for testing (see Table S2 in the supplemental material) were produced centrally at the NIBSC laboratory and distributed to participating laboratories as common antigens or produced in-house in individual laboratories from virus stocks of nominally the same viruses. Egg virus stocks were all grown in 10- to 11-day-old embryonated hen eggs. Cell viruses were propagated in MDCK cells (H1N1, B viruses) or SIAT MDCK cells (H3N2).

10.1128/mSphere.00567-21.8 TABLE S2 Common source influenza virus strains used in the studies. Download Table S2, DOCX file, 0.01 MB. Copyright © 2021 Waldock et al. 2021 Waldock et al. https://creativecommons.org/licenses/by/4.0/ This content is distributed under the terms of the Creative Commons Attribution 4.0 International license.

### Common source reagents.

Each laboratory was provided with seed viruses for growing in-house stocks of antigen (provided by NIBSC), common source antigens for FLUCOP testing (produced at NIBSC), and a common stock of RDE for FLUCOP testing (Denka Seiken, provided by SP).

### Serum panels.

Four serum panels were used in the study: panel 1 was used in the initial DOE, aimed at the development of the consensus SOP, and panel 2, the confirmatory study. Both panels included human pre- and postvaccination samples (*n* = 30 [15 pre-/postvaccination pairs] for panel 1; *n* = 30 [6 pairs and 18 nonpaired {3 pre- and 15 postvaccination}] for panel 2), 3 HAI pooled human serum samples, duplicate samples of the international H1N1 pdm09 standard, and 5 control samples. Control samples included two positive sheep serum controls (sheep control 1, prepared with vaccine-specific strains, and sheep control 2, which included one strain that was different from the vaccine strain, although from the same virus subtype), an Ig-depleted serum (Sigma-Aldrich S5393) and two in-house controls (IQC 1 and IQC 2). Serum panel 2 did not include Ig-depleted serum and international H1N1 standard but included additional strain-specific ferret serum samples. Panel 3 was used for testing WT egg- and cell-grown viruses: *n* = 20 (2 paired and 16 nonpaired [3 pre- and 13 postvaccination] human serum samples). Panel 4 was used for testing WT cell-grown H3N2 with and without oseltamivir: *n* = 15 (2 paired and 11 unpaired [2 pre- and 9 postvaccination] human serum samples). All human serum samples were obtained from SP (SeraCare 2015–2016 trivalent influenza vaccinated [TIV] [A/California/07/2009, A/South Australia/55/2014, B/Phuket/3073/13], 2015–2016 quadrivalent influenza vaccinated [QIV] [A/California/07/2009, A/South Australia/55/2014, B/Phuket/3073/2013, B/Brisbane/60/2008]) and NIBSC (2015–2016 TIV [split virion] from Sanofi Pasteur, containing A/California/07/2009, A/Switzerland/9715293/2013. and B/Phuket/3073/2013).

All sheep and ferret sera were provided by NIBSC. Sera were aliquoted centrally and randomized for each participating laboratory, using the SP central sample management, and then distributed to each laboratory.

### Biological standards.

The following biological standards were tested in the confirmatory study: sheep sera from hyperimmunized animals, ferret sera from infected ferrets, three pools of postvaccination human blood donations, and the 2nd International Standard for antibody to influenza H1N1pdm09 virus (NIBSC code 10/202). The sheep and ferret sera were from animals immunized or infected with the same influenza virus strains as those tested in the current study. Human sera were taken from three different influenza vaccination campaigns, 2010–2011, 2013–2014, and 2015–2016 (Table 2); the strains tested in the current study matched those used in the most recent vaccination campaign (2015–2016).

### Ethics statement.

Informed consent was given to use these samples for research purposes. No additional consent was required from sample donors for use of their samples in this study. All husbandry and procedural work were conducted under UK Home Office licenses and approved by NIBSC’s Animal Welfare and Ethics Review Body (AWERB).

### Data analysis.

Results from all laboratories were compiled, independently checked by two separate FLUCOP partners, and analyzed using both a classical statistical method (mixed model) and mathematical method based on a proprietary algorithm of data analysis developed by Quinten (Q-finder). Data consisted of replicate titers expressed as the reciprocal of serum dilutions. Titers of <10 were assigned a value of 5 for calculations. All 6 laboratories returned a full data set for the first serum panel tested. One laboratory returned singleton titers for in-house testing using serum panel 2, and these were excluded from further analysis.

### (i) Estimation of interlaboratory and interrun variation using mixed model statistics.

To evaluate the laboratory-to-laboratory variation, the geometric mean of the replicates was calculated, thereby eliminating intralaboratory variation. Interlaboratory variation for each of the run conditions was calculated as the standard deviation of the log_2_ geometric HAI titer of the combined replicates for each sample across the six laboratories.

### (ii) Estimation of the impact of assay parameters on %GCV and GMTs using mixed model statistics.

To evaluate the impact of each experimental parameter on assay variability (%GCV) and HAI titers (GMT), the following mixed effect models were conducted on the standard deviation (SD) of individual samples and the log_2_-transformed HAI titers for each influenza virus strain. GCV was calculated from the SD by the following formula: $\text{GCV = 100 × }\sqrt{e^{{\text{var}}_{\text{ln}}}\;-\;1}$, where var_ln_ = (SD_log2_ × ln 2)^2^.

$$\text{SD}=X\beta \;+{\text{ε}}_{\text{Sample}}+\text{ε},\text{log}\left(\text{titer}\right)=X\beta \;+{\text{ε}}_{\text{Sample}}\;+\;{\text{ε}}_{\text{Lab|Sample}}\;+\text{ε}$$

*X* is the design matrix of the 6 fixed experimental parameters that include the age of RBC, the concentration of RBC, and the time and temperature of first incubation and second incubation. β is the vector of the coefficients (impact) of each experimental parameter. In the model of variability (SD), ε_Sample_ is the random factor of the distribution of the sample panel ${\text{ε}}_{\text{Sample}}\text{ \textasciitilde{} }N\left(0,\sigma_{\text{SD,Sample}}^{\text{2}}\right)\text{,iid}$, while ε is the uncontrolled error$\text{ε \textasciitilde{} }N\left(0,\sigma_{\text{SD, error}}^{\text{2}}\right)\text{, iid}$. In the model of HAI titers, ε_Sample_, ε_Lab/Sample_, ε are random effects that follow the distribution ${\text{ε}}_{\text{Sample}}\text{ \textasciitilde{} }N\left(0,\sigma_{\text{Titer, Sample}}^{\text{2}}\right)\text{, iid, ε Lab|Sample}\text{ \textasciitilde{} }N\left(0,\sigma_{\text{Titer, Lab|Sample}}^{\text{2}}\right)\text{,iid}$, and $\text{ε \textasciitilde{} }N\left(0,\sigma_{\text{Titer, error}}^{\text{2}}\right)\text{, iid}$. Notice the laboratory is modeled as a random effect nested under sample panel in the HAI titer model.

### (iii) Normalization.

HAI titers were normalized by dividing the observed HAI titer by the normalizer titer obtained per lab and run condition. These values were subsequently multiplied by the geometric mean normalizer titer calculated per run condition across labs.

### (iv) Mathematical analysis with Q-finder.

Low variability between participating laboratories was defined as (i) at least 4 out of the 6 testing laboratories having a maximum 2-fold difference between sample duplicates and (ii) a maximum of 2-fold difference in titer being observed between laboratories that satisfy criterion 1. High or low variability was calculated for each strain, for each condition tested, and for each sample. The Q-finder algorithm was used to identify combinations of parameters (incubation temperatures, incubation times, and concentration and age of RBCs) that are highly associated with low variability between laboratories and to potentially identify combinations of conditions that may further lower interlaboratory variation that were not tested in the study.

Q-finder is a proprietary supervised learning algorithm. No particular assumption is made regarding the shapes of distribution of the outcome or explanatory variables. The outcome comprises several classes (e.g., low/high variability), and the algorithm explores the space of explanatory variables to identify areas of overconcentration of the class of interest specified for the exploration (here, low variability). All combinations of variables (categorical and continuous) are systematically explored. The output is a set of rules, a rule being defined as a combination of variable modalities that characterizes a subgroup of conditions. The variables in a rule are either continuous variables defined by lower and upper bounds or categories for qualitative variables. The maximum number of variables per rule is generally set to 3 to keep them understandable. For the sake of interpretation, only “open” rules (rules in the form ≥A or ≤B) are retained. Rules are selected based on their size and hypergeometric *P* value. The size of a rule refers to the number of conditions satisfying the rule criteria. The *P* value is defined as the probability of obtaining by chance a rule of a given size with a given proportion of the class of interest:

$$P_{\text{hypergeometric}}\left(k,\;r,\;n,\;N\right)=1\;-\;\overset{k-1}{\underset{i={\text{min}}_{X}}{\sum }}P\left(X\;=i\right)$$

for which *k* is the number of conditions in the class of interest in the rule, *r* is the number of conditions in the class of interest in the full data set, *n* is the number of conditions in the rule, *N* is the number of conditions in the full data set, min*_X_* is the maximum between 0 and *r* + *n*  −  *N*, and *P*(*X* = *k*) is the probability of obtaining a rule defined by (*k*, *r*, *n*, *N*) as follows:

$$P\left(x=k\right)=\frac{\left(\begin{array}{c} r\\ k \end{array}\right)\left(\begin{array}{c} N\;-\;r\\ n\;-\;k \end{array}\right)}{\left(\begin{array}{c} N\\ n \end{array}\right)}$$
