# Supplementary material for: Assay Harmonization and Use of Biological Standards To Improve the Reproducibility of the Hemagglutination Inhibition Assay: a FLUCOP Collaborative Study
Source: mSphere. 2021 Jul 28;6(4):e00567-21. doi: 10.1128/mSphere.00567-21 (PMC8530177; doi:10.1128/mSphere.00567-21)

FLUCOP

Standardization and Development of Assays for Influenza Vaccine Correlates of Protection

Haemagglutination Inhibition Assay (Turkey Red Blood Cells)

FluCOP-WP#1 HAI SOP-2

Consensus protocol for collaborative study (TRBCs)

March 2019

TABLE of CONTENT

INTRODUCTION 3

1 - EQUIPMENTS 3

2 - HANDLING AND PREPARATION OF SAMPLES (prior to HAI assay) 4

3 - OPERATING PROCEDURE 4

3-1 Preparation of RBCs and serum samples 4

3.1.1 Preparation of Turkey Red Blood Cell (TRBC) suspensions 4

3.1.1.1 Washing of the whole turkey blood 4

3.1.1.2 Preparation of a 15% TRBC suspension – for red blood cell adsorption of sera samples only 4

3.1.1.3 Preparation of a 0.5% TRBC suspension – for use in the HAI assay 5

3.1.2 Preparation of Serum Samples 5

3.1.2.1. RDE treatment 5

3.1.2.2. TRBC adsorption of sera 5

3.2 HA virus titration 6

3.3 Virus dilution and Back titration 7

3.3.1 Virus dilution to 4HAU/25µL 7

3.3.2 Virus back titration 7

3.4 HI Sera samples Titration 9

3.4.1 Sera dilution (large cohort where sera are diluted 1/10) 9

3.4.2 Sera dilution (small cohort where sera are diluted 1/5) 10

3.4.3 Virus addition 11

3.4.4 TRBCs addition 11

3.4.5 Reading 11

3.4.6 Quality control and validity criteria 11

INTRODUCTION

Haemagglutination (HA) describes the binding of sialic acid receptors on red blood cells (RBCs) by influenza viruses causing a lattice formation of cells within solution. When using RBCs, haemagglutination can be easily visualised as free RBCs drop out of solution and settle at the bottom of a microplate well. The HA titer of the virus (a measure of the amount of HA-binding virus in the sample) is determined by serially diluting virus and mixing with RBCs. The reciprocal of the final dilution for which complete haemagglutination still occurs is the HA titre of that virus sample. To measure influenza specific antibodies in serum samples the same principle can be used. A defined amount of virus is incubated with serum, allowing specific antibodies to bind virus. Subsequently RBC are added – where antibodies are present and bound to the virus, RBCs are no longer agglutinated, and will fall out of solution. Serially diluting the serum sample, mixing with a fixed amount of virus and RBCs allows an endpoint to be determined – this is the reciprocal of the final serum dilution where complete inhibition of agglutination occurs, or the HI or HAI titre of that serum sample.

1 - EQUIPMENTS

Protective equipment and clothing	***Working equipment and materials***	***Reagents and Disinfectant***
Microbiological safety cabinet<br>Disposable gloves<br>Spray bottle for ethanol/decontaminant<br>Autoclave bag<br>Autoclave tape<br>Tip disposal container<br>Autoclave boxes	Water or heat bath @ 56°C and 37°C<br>Incubator @37°C<br>Pipetboy or equivalent<br>Refrigerator @ +5°C ±3°C (Bench)<br>Refrigerated centrifuge<br>96 well V bottom microtitre plates (Greiner #G651101 or equivalent)<br>Gilson pipettes or equivalent<br>Multichannel pipette<br>Pipette tips<br>50 mL centrifuge tubes<br>Plastic pipettes<br>Vortex<br>Timer	**Turkey RBCs**, stored as whole blood PBS (without Calcium and Magnesium), pH7.1-7.4 (for example Sigma #D8537 or equivalent)<br><br>Receptor destroying enzyme (RDE) (Denka)<br><br>Sterile water<br><br>70% ethanol

2 - HANDLING AND PREPARATION OF SAMPLES (prior to HAI assay)

Serum samples should remain frozen at -70°C until use. Samples should be thawed to room temperature and treated with RDE ± turkey red blood cells prior to use in the assay (described below).

Virus should be stored at -70°C. Larger volumes of virus should be thawed at +5°C ±3°C overnight, and 0.5mL aliquots made and stored at -70°C. A new aliquot of virus should be thawed for each HI assay on the day of use.

Live virus should be handled at BSL2 level in accordance with in-house SOPs.

Turkey Red Blood Cells (TRBCs) should be stored as whole blood or packed TRBCs at +5°C ±3°C.

3 - OPERATING PROCEDURE

3-1 Preparation of RBCs and serum samples

3.1.1 Preparation of Turkey Red Blood Cell (TRBC) suspensions

Turkey whole blood should be stored at +5°C ±3°C for up to 7 days post bleeding. Washing will be performed to prepare packed or needed red blood cell preparation on the day of use.

3.1.1.1 Washing of the whole turkey blood

- Centrifuge the whole turkey blood for 10 minutes at 500g (+5°C ±3°C) and discard the supernatant
- Gently re-suspend the TRBCs in PBS, centrifuge for 5 minutes at 500g (+5°C ±3°C) and discard the supernatant
- Repeat the preceeding step
- Gently re-suspend the TRBCs in PBS and centrifuge for 10 minutes at 500g (+5°C ±3°C) to pellet the cells
- Discard carefully the supernatant to obtain **packed TRBCs**

Packed TRBCs can be stored at 2-8°C for up to 7 days post bleeding.

3.1.1.2 Preparation of a 15% TRBC suspension – for red blood cell adsorption of sera samples only

Using a Gilson (or equivalent) pipette take up the volume of packed TRBCs required to make the correct percentage of TRBCs in PBS e.g. add 750µL of packed red blood cells to 5 mL of PBS. As packed red blood cells are viscous, careful and slow pipetting is required. Gently mix the TRBCs and PBS by inverting until TRBCs are evenly distributed in solution – *rapid mixing or shaking can result in lysis of TRBCs*

Prepared 15% TRBC suspension should be used on the day of preparation.

3.1.1.3 Preparation of a 0.5% TRBC suspension – for use in the HAI assay

- Using a Gilson (or equivalent) pipette take up the volume of packed TRBCs required to make the correct percentage of TRBCs in PBS e.g. for a 0.5% solution in a 50mL centrifuge tube, add 250µL of packed red blood cells to 50mL of PBS. As packed red blood cells are viscous, careful and slow pipetting is required.
- Gently mix the TRBCs and PBS by inverting until TRBCs are evenly distributed in solution – rapid mixing or shaking can result in lysis of TRBCs

Prepared 0.5% TRBC suspensions should be used on the day of preparation.

3.1.2 Preparation of Serum Samples

All serum samples must be heat-inactivated at 56°C for 30min and then treated with receptor destroying enzyme (Denka RDE) to remove non-specific inhibitors of haemagglutination present in serum samples.

3.1.2.1. RDE treatment

- Prepare RDE according to the manufacturer's instructions: reconstitute the product in 20mL of sterile water
- Dilute serum sample by mixing 1 part sera and 4 parts prepared RDE (resulting in a 1/5 dilution of the sera) e.g. add 50µL of sera to 200 µL of RDE
- Homogenize with a vortex
- Incubate for 18h at 37°C either in an incubator or a heat/water bath
- Heat inactivate at 56°C for 60 minutes
- Place RDE treated sera at +5°C ±3°C

RDE treated sera can be stored at +5°C ±3°C for 14 days, or for long term storage ≤20°C. It is recommended to store aliquots of RDE treated sera at -20°C.

3.1.2.2. TRBC adsorption of sera

After RDE treatment, TRBC adsorption of the sera must be carried out to remove non-specific agglutinins. For large cohorts, e.g. clinical trial samples, RDE treated sera samples will be treated with a 15% TRBC suspension whereas for small cohorts, only sera samples showing non-specific agglutination will be further treated with packed red blood cells

TRBC adsorption of the sera with <u>15% TRBC suspension</u> (large cohorts)

- Add 1 part of 15 % TRBC suspension to 1 part of RDE treated sera, and mix gently by inversion, e.g. add 250µL of 15% TRBC suspension to 250 µL of RDE treated sera (containing 50 µL of neat serum and 200µL of RDE)
- Incubate for 30 minutes at room temperature
- Centrifuge at 13krpm (or the top speed of a small benchtop) for 3 minutes to pellet the red blood cells and transfer the serum to a clean tube or a 96 deep well plate and seal the plate. Tubes or plates of RDE and RBC absorbed sera can be stored at -20°C for as long as required or +5°C ±3°C for 14 day. Discard the pelleted red blood cells.

Caution: the sera are now diluted at 1:10

TRBC adsorption of the sera with packed RBCs (Small cohorts)

For small cohorts of samplesonly serum showing non-specific agglutination will be treated with packed red blood cells. Once serum samples have been RDE treated they should be assessed for the presence of non-specific agglutinins:

- Add 25µL of PBS to one row of a V-bottomed plate (in landscape format) for every serum sample to be tested and in one row for the TRBC control (TRBCs+PBS only)
- Add 25µL of the serum sample to the first well (A1 for the first sample, B1 for the second and so on…) and perform 2-fold dilutions by transferring 25µL from wells 1 to 12
- Gently re-suspend the 0.5% TRBC suspension by inverting and add 25µL in all the wells
- Gently shake and cover the plate
- After 30 minutes at room temperature, read the plate after tilting and record any non-specific agglutination – as no virus is present all TRBCs should fall out of suspension and form a button. Note any serum samples where a reddish carpet is seen and TRBCs have been agglutinated.

These samples (showing non-specific agglutination) will need to be treated with packed red blood cells to remove them as described below:

- Add 1 part of packed TRBCs to 19 parts of RDE treated serum and mix gently by inversion, e.g. add 10 µL of packed red blood cells to 190 µL of RDE treated sera
- Incubate at room temperature for 30 minutes
- Centrifuge at 13krpm (or top speed of a small benchtop centrifuge) for 3 minutes to pellet TRBCs and transfer the serum to a clean tube. Discard the pelleted TRBCs.

Caution: RDE treated sera or RDE-packed TRBCs treated sera are diluted at 1:5.

3.2 <u>HA virus titration</u>

- In a V-bottomed plate (in landscape format), using a multichannel pipette, add 50µL of PBS to one row for each virus to be tested, including one row for the TRBC control (TRBCs+PBS only)
- Add 50µL of virus to the first well - virus starting dilution is ½ (A1 for the first virus, B1 for the second and so on…)
- Perform 2-fold dilutions from wells 1 to 12 - using a multichannel pipette mix the virus + PBS in the first column, transfer 50µL to the next column and mix. Repeat across the plate. Discard the remaining 50µL after mixing in column 12
- Gently re-suspend the 0.5% TRBC suspension and add 50µL to each well
- Gently shake and cover the plate
- Incubate at RT for 30 minutes
- Tilt and read the HA titre as the reciprocal of the highest dilution where complete haemagglutination still occurs i.e. a reddish carpet of agglutinated cells where RBC do not stream (see Figure 1). The red blood cell wells are used as reference for behaviour of non-agglutinated red blood cells.

Caution: the titer is expressed in HAU/50µL

3.3 Virus dilution and Back titration

3.3.1 Virus dilution to 4HAU/25µL

- Calculate the dilution factor by dividing the HA titre by 8 (as 50µL of virus are used to ascertain the HA titre of the virus, this is 8HAU in 50µL, equivalent to 4HAU in 25µL). Calculate the volume of antigen (virus) required by dividing the total required volume by the dilution factor
- Calculate the volume of PBS required by subtracting the volume of antigen (virus) from the total volume required
- Mix the required volume of PBS and antigen (virus) by vortexing

E.g. 2.4mL of antigen (virus) are required for each 96 well plate (+ ~10% for overage = 2.7mL). To calculate the dilution for 10 plates, 27mL in total:

Antigen (Virus)	HA titre	Dilution factor	Antigen (µL)	PBS (mL)
Example 1	1280	160	169	26.831
Example 2	320	40	675	26.325

3.3.2 Virus back titration

Confirm the virus dilution is 4HAU in 25µL performing a back titration (4 replicates for each virus)

- In a V-bottomed plate (in landscape format), add 25µL of PBS (excluding the first column) to four rows for each virus to be tested i.e. wells 2-12 (see Fig 2A)
- Add 25µL of diluted antigen (virus) to the first two wells in each of the four rows
- Perform 2-fold dilutions from wells 2 to 6 – the back titration will assess undiluted antigen (1/1), 1/2 dilution, 1/4, 1/8, 1/16 and 1/32 only. Discard the remaining 25µL after mixing well 6. Wells 7-12 are for TRBC only controls (see Figure 2A and 2B).
- Add 25µL of PBS in all the wells
- Gently re-suspend the 0.5% TRBC suspension and add 50µL to each well
- Gently shake and cover the plate
- Incubate at room temperature for 30 minutes
- Tilt the plate to read. See Figure 2C for guidelines on reading the back titration. TRBCs should run to the bottom of the well in the 1/8, 1/16 and 1/32 dilution, but should still be agglutinated in the 1/1, 1/2 and 1/4 dilutions. TRBC only wells are used as reference for behaviour of non-agglutinated TRBCs.
- If 4 HAU/25µL is not achieved the dilution must be adjusted and a second back titration carried out. If the first back titration shows 2 HAU in 25µL, meaning virus has been diluted too much, add the calculated volume of antigen again to your virus dilution. If the back titration shows 8HAU in 25µL, meaning the virus has not be diluted enough, add the calculated volume of PBS again to your virus dilution. Repeat the preceeding steps until 4 HAU in 25µL is achieved.

e.g. in this example after 1 adjustment the required 4HAU/25µL is reached. Sometimes several adjustments are needed.

Virus	Antigen (µL)	PBS (mL)	HAU/25µL in 1st back titration	Adjustment to the dilution	HAU/25µL in 2nd back titration
Example 1	169	26.831	2	169 µL antigen added	4
Example 2	675	26.325	8	26.325 PBS added	4

3.4 HI Sera samples Titration

3.4.1 Sera dilution (large cohort where sera are diluted 1/10)

- In a V-bottomed plate (in landscape format), add 25µL of PBS to a wells 2-12 in one row for each sera sample to be tested. The number of plates required is the (number of sera X the number of virus)/8. 8 sera/virus combinations, or 7 in case of an optional row of control red blood cells, can be run on a single plate with one column for a serum control (see Figure 3). Any control/standard sera should be included in the calculation as it is not necessary to run these on every plate, a single control/standard row within a batch/experiment is sufficient.
- Add 25µL of RDE-15% TRBC suspension treated sera into the first, the second and the last well of a row – A1, A2 and A12 for the first serum sample, B1, B2 and B12 for the second and so on... Repeat for all viruses to be tested. *Remember clean tips are essential to avoid cross contamination*
- Using a multichannel pipette, perform a 2-fold dilution across the plate from wells 2 to 11 – mix the sera and PBS in well 2 and transfer 25µL to well 3, mix and repeat across the plate. Discard the remaining 25µL after mixing well 11.

  Clean tips are not required between transfers as carryover is assumed to be minimal

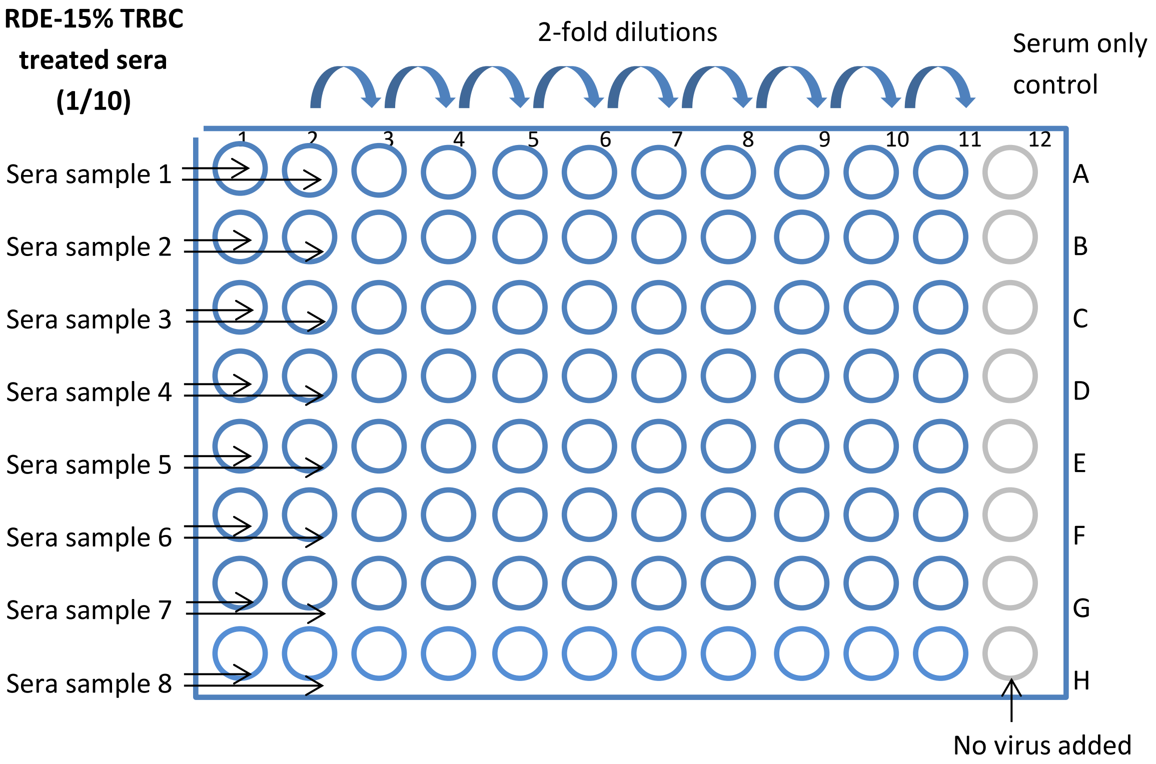

3.4.2 Sera dilution (small cohort where sera are diluted 1/5)

- In a V-bottomed plate (in landscape format), add 25µL of PBS to a wells 1-12 in one row for each sera sample to be tested. The number of plates required is the (number of sera X the number of virus)/8. 8 sera/virus combinations, or 7 in case of an optional row of control red blood cells, can be run on a single plate with one column for a serum control (see Figure 3). Any control/standard sera should be included in the calculation as it is not necessary to run these on every plate, a single control/standard row within a batch/experiment is sufficient.
- Add 25µL of RDE-15% TRBC suspension treated sera into the first and the last well of a row – A1 and A12 for the first serum sample, B1 and B12 for the second and so on... Repeat for all viruses to be tested. *Remember clean tips are essential to avoid cross contamination*
- Using a multichannel pipette, perform a 2-fold dilution across the plate from wells 1 to 11 – mix the sera and PBS in well 1 and transfer 25µL to well 2, mix and repeat across the plate. Discard the remaining 25µL after mixing well 11.

  Clean tips are not required between transfers as carryover is assumed to be minimal

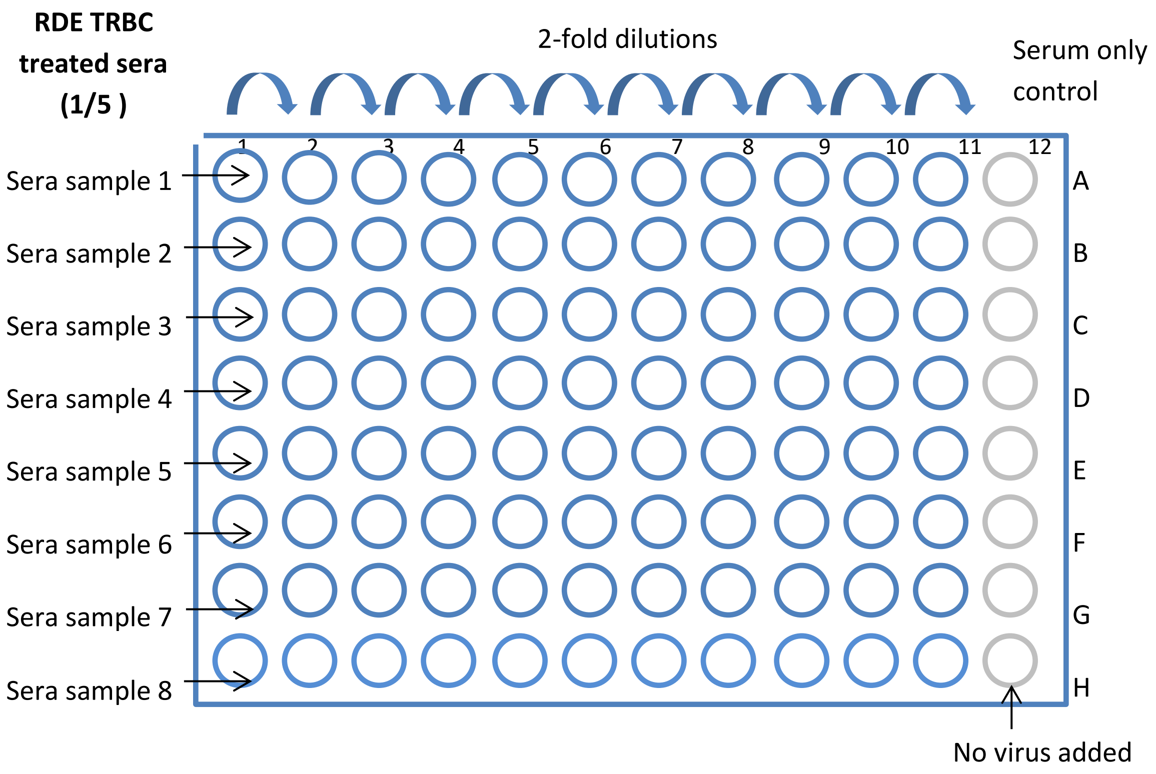

3.4.3 Virus addition

- Add 25µL of 4HAU/25µL virus to each well of the plate except in column 12 (serum controls). In case of the presence of a TRBC control distribute 25µL of PBS instead of virus.
- Gently shake and cover the plate
- Incubate at room temperature (record temp on results sheet) for **90 minutes**

3.4.4 TRBCs addition

- Gently re-suspend 0.5% TRBC suspension by inverting
- Add 50µL of TRBC to each well of each plate
- Incubate at room temperature (record temp on results sheet) for 30 minutes

3.4.5 Reading

see Figure 3 for guidelines on reading HAI titres

- Tilt the plate to read the samples and the control in column 12 simultaneously. Allow the TRBCs to run to the bottom of the well in column 12. The control serum wells in column 12 are used as reference for behaviour of non-agglutinated red blood cells.
- The HAI titre is defined as the reciprocal of the highest dilution of the test sample where complete inhibition of agglutination occurs i.e. where the TRBCs run to the bottom of the well.

3.4.6 Quality control and validity criteria

Each serum sample is tested in two independent assay runs (generated from two independent antigen dilutions and two independently RDE treated serum aliquots). The two replicate HAI titres of the same sample must be within 2-fold difference between each other. If HAI titre differences are greater than 2-fold a third replicate is carried out.

Figure 1. Reading haemagglutination titres. *Example of reading the HA titre for a A/H1N1 virus. The reciprocal of the highest virus dilution where complete agglutination still occurs is given as the HA titre. In this example the HA titre of the H1N1 virus is 640.*

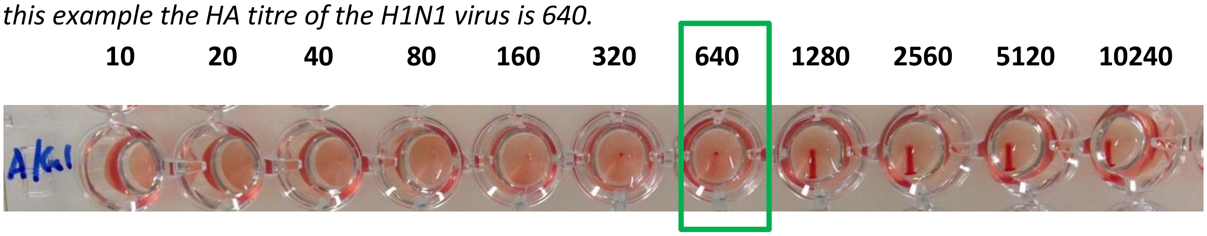

Figure 2. Setup of virus back titration plate and guidance on reading back titrations

2A. *Four replicates of virus #1 back titration (Row A to Row D) and potentially virus #2 (Row E to Row H)*

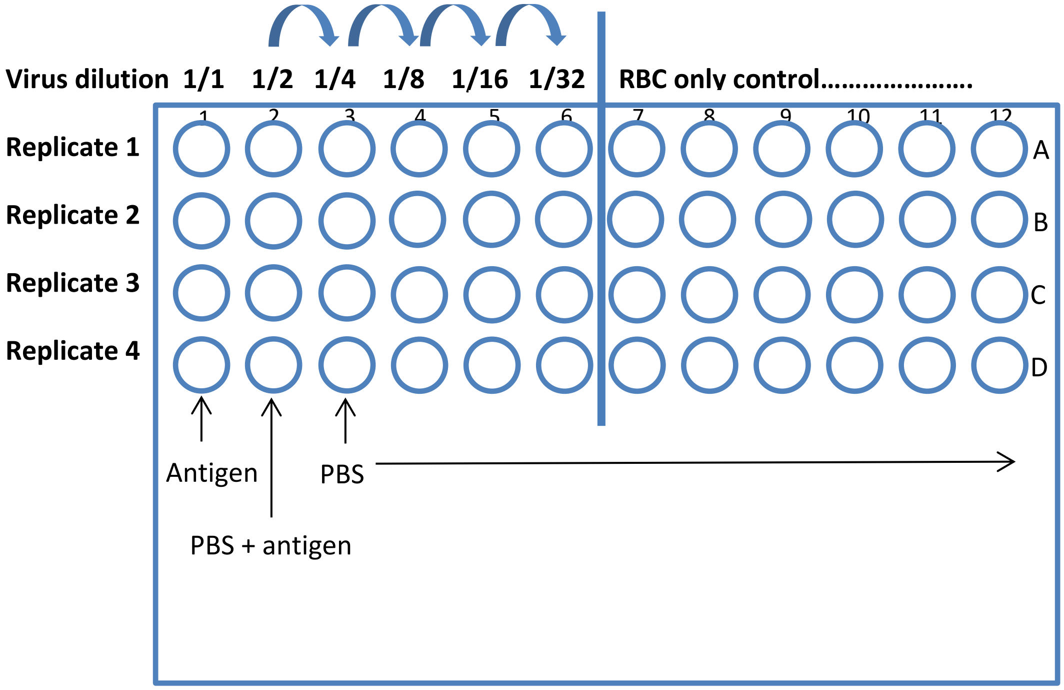

2B. Example of back titration of two viruses. Both virus #1 (A-D) and virus #2 (E-H) show a back titration of 4 HAU/25µL

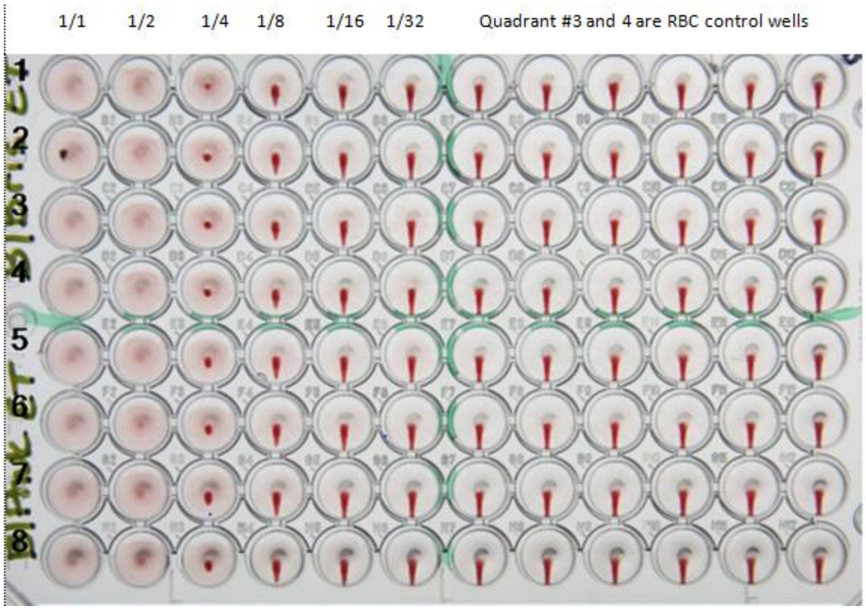

2C. Guidance on assigning back titration titres. The cartoon gives a range of examples of 2-8 HAU/25µL.

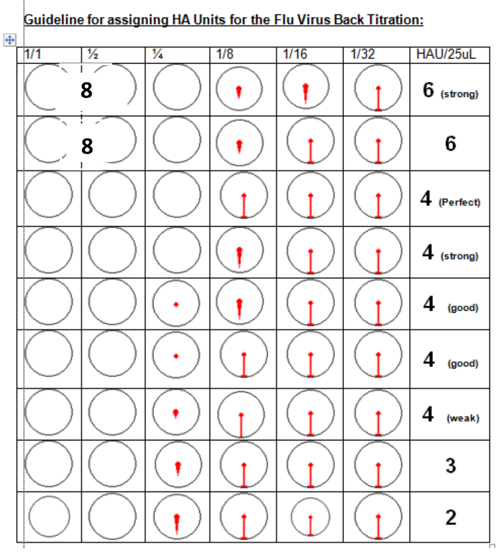

Figure 3. A. Example of an H1N1 HAI plate B. Guidelines on reading HAI: example of plate. Yellow circles show the serum only control – expect to see no agglutination of TRBCs. Red circles show agglutination of TRBCs. Blue circles show complete inhibition of agglutination and green circles show the highest dilution where agglutination is still inhibited – the reciprocal of this is the HAI titre.

A.

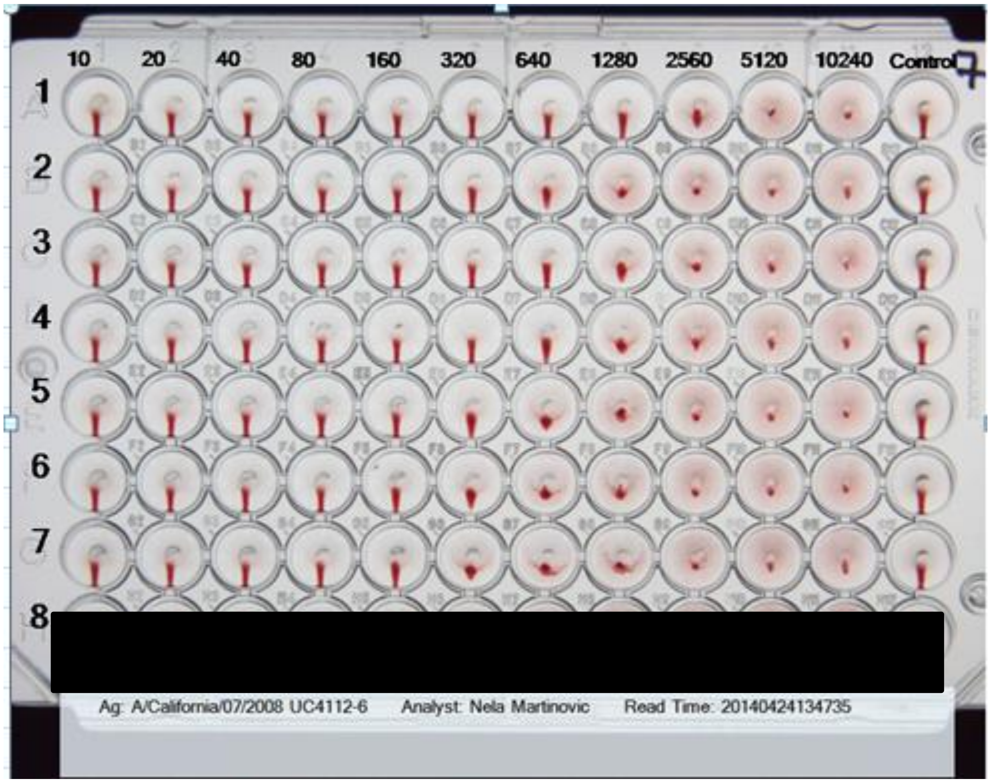

B.

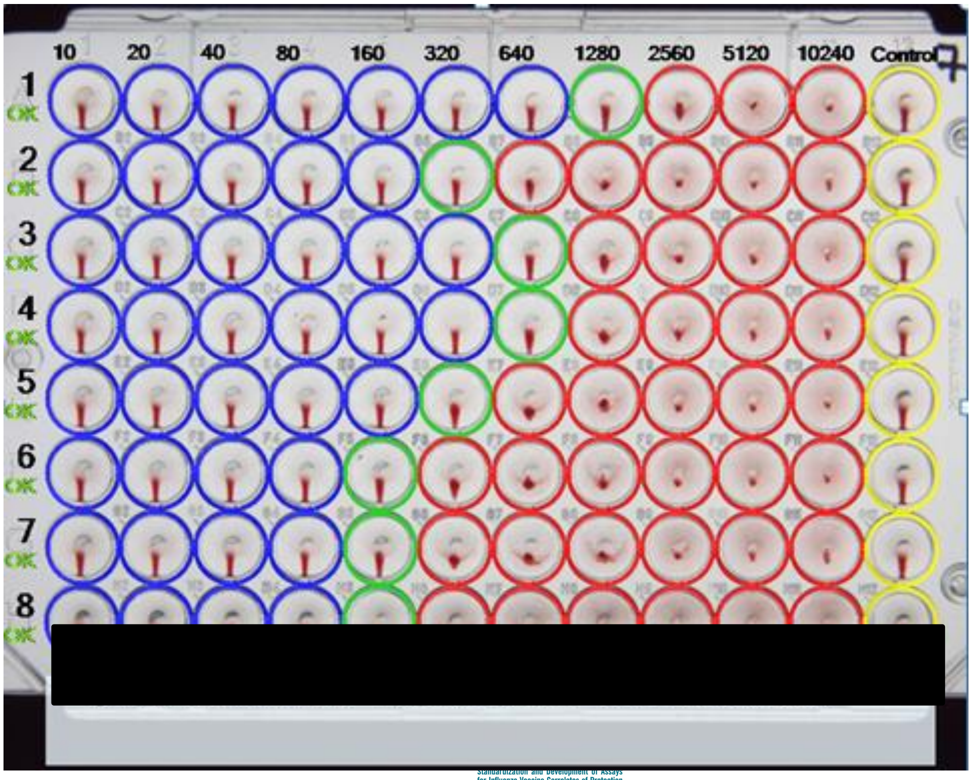

Supplement: TEXT S1 [file msphere.00567-21-s0001.pdf]
