# Supplementary figures and images for: Assay Harmonization and Use of Biological Standards To Improve the Reproducibility of the Hemagglutination Inhibition Assay: a FLUCOP Collaborative Study
Source: mSphere. 2021 Jul 28;6(4):e00567-21. doi: 10.1128/mSphere.00567-21 (PMC8530177; doi:10.1128/mSphere.00567-21)

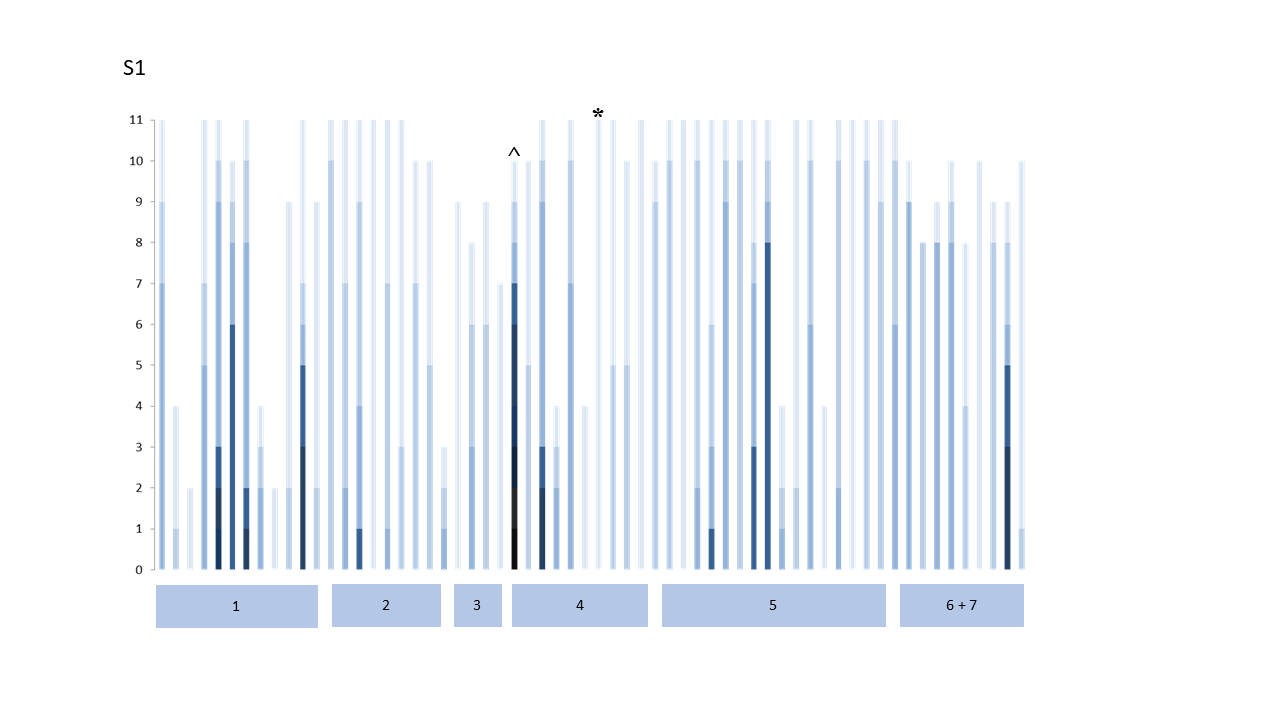

Supplement: FIG S1 [file msphere.00567-21-sf001.tif]

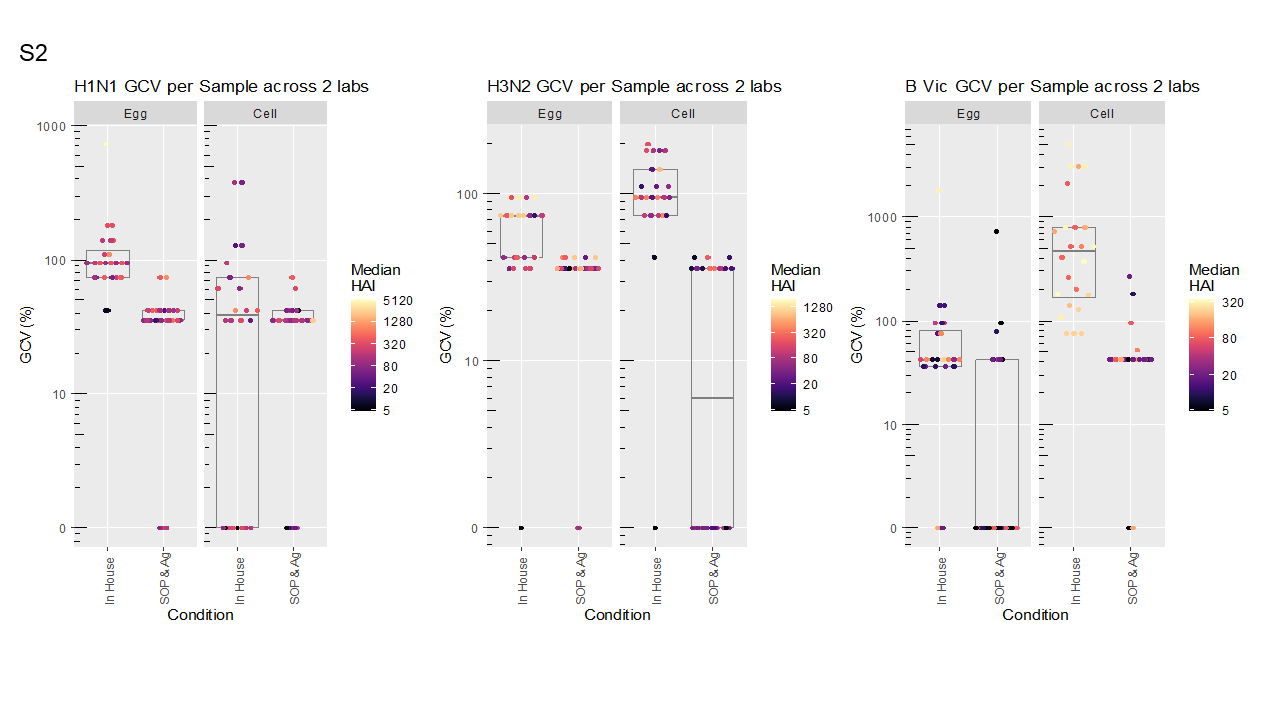

Supplement: FIG S2 [file msphere.00567-21-sf002.tif]

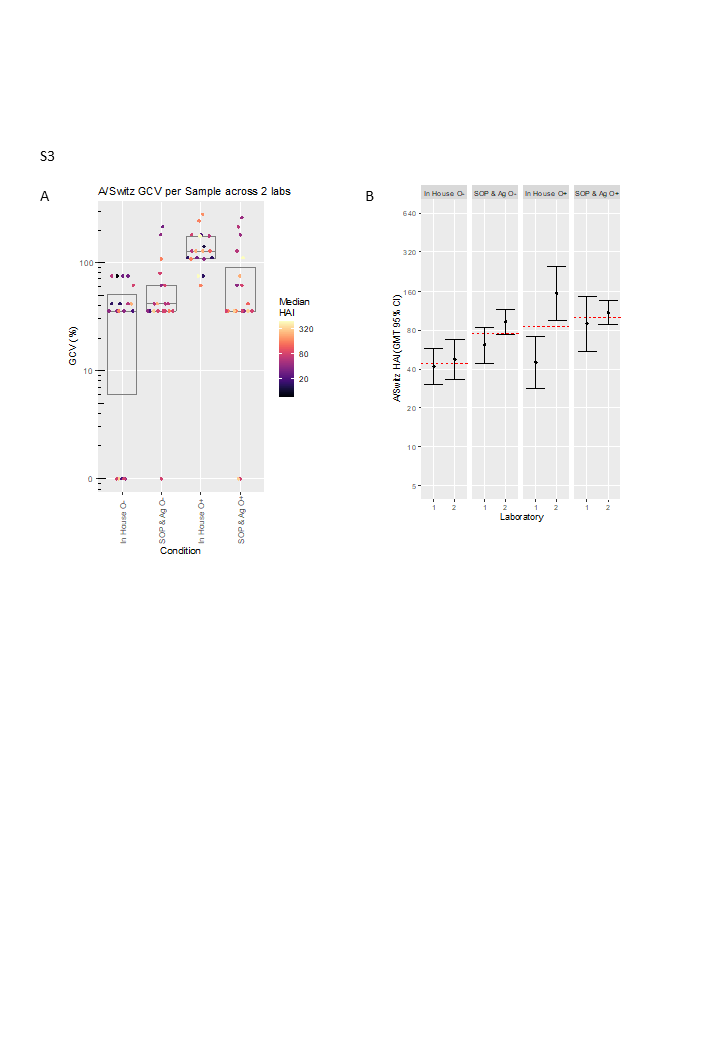

Supplement: FIG S3 [file msphere.00567-21-sf003.tif]

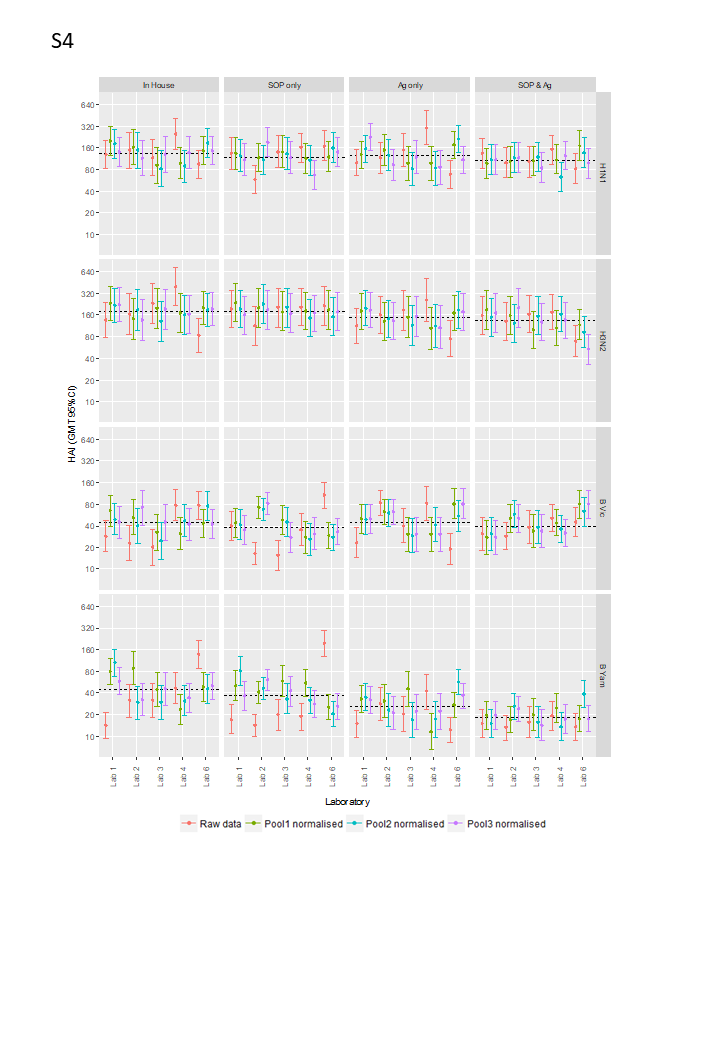

Supplement: FIG S4 [file msphere.00567-21-sf004.tif]

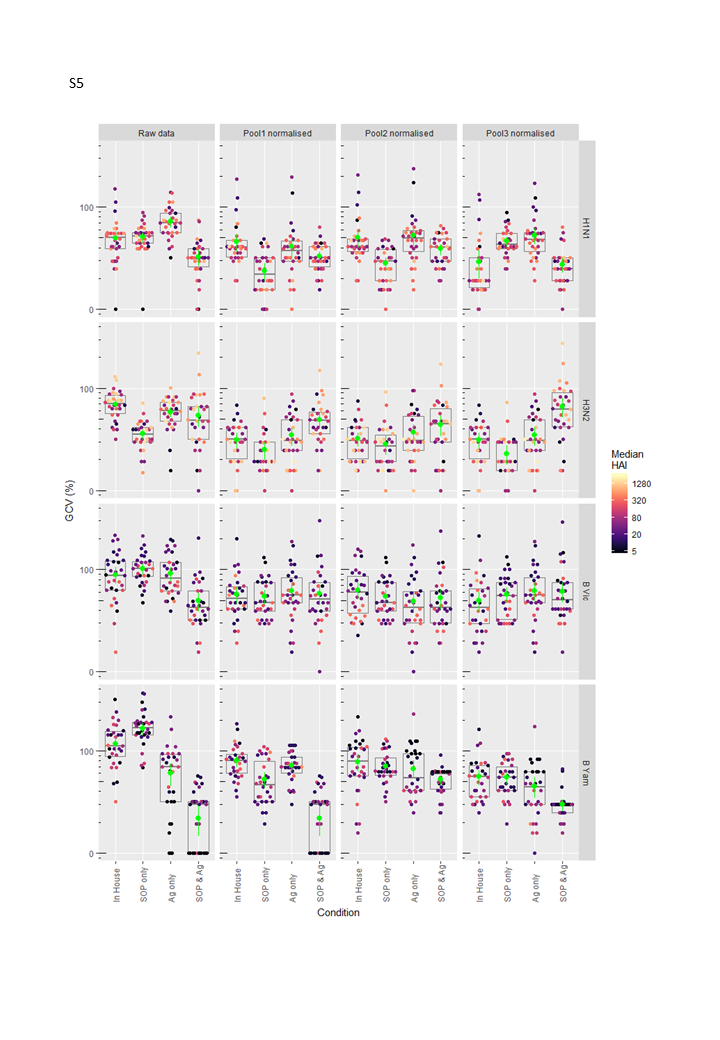

Supplement: FIG S5 [file msphere.00567-21-sf005.tif]
