# Supplementary material for: Assay Harmonization and Use of Biological Standards To Improve the Reproducibility of the Hemagglutination Inhibition Assay: a FLUCOP Collaborative Study
Source: mSphere. 2021 Jul 28;6(4):e00567-21. doi: 10.1128/mSphere.00567-21 (PMC8530177; doi:10.1128/mSphere.00567-21)
Supplement: TABLE S1 [file msphere.00567-21-st001.docx]

**Supplementary Table S1** Parameter settings, for analysis in a fractional factorial DOE.

| **Parameter** | **Setting** | | |
| --- | --- | --- | --- |
|  | **Low, –1** | **Normal, 0** | **High, 1** |
| Age of RBCs, days | 1–5  (Fresh) | – | 7–11  (Old) |
| Concentration of RBCs, % | 0.35 | 0.50 | 1.00 |
| Incubation 1 time, min | 30 | 60 | 90 |
| Incubation 1 temperature, °C | RT | – | 37 |
| Incubation 2 time, min | 30 | 45 | 60 |
| Incubation 2 temperature, °C | RT | – | 37 |

DOE, design of experiment; RBC, red blood cell; RT, room temperature

Incubation 1, virus + serum; incubation 2, virus + serum+ red blood cells
