# Supplementary material for: Assay Harmonization and Use of Biological Standards To Improve the Reproducibility of the Hemagglutination Inhibition Assay: a FLUCOP Collaborative Study
Source: mSphere. 2021 Jul 28;6(4):e00567-21. doi: 10.1128/mSphere.00567-21 (PMC8530177; doi:10.1128/mSphere.00567-21)
Supplement: TABLE S2 [file msphere.00567-21-st002.docx]

**Supplementary Table S2. Common source influenza virus strains used in the studies**.

| **Virus** | **Reassortant/WT** | **HA titre (TRBCs)** |
| --- | --- | --- |
| A/California/7/2009 NYMC X-181 (H1N1), | Reassortant Egg | 512 |
| A/Switzerland/9715293/2013 NIB-88 (H3N2), | Reassortant Egg | 512 |
| B/Brisbane/60/2008 NYMC BX-35 (B/Victoria lineage) | Reassortant Egg | 512 |
| B/California/12/2015 NYMC BX-59A (B/Yamagata lineage). | Reassortant Egg | 256 |
| A/California/07/2009 (H1N1) | WT Egg | 128 |
| A/Switzerland/9715293/2013 (H3N2) | WT Egg | 256 |
| A/California/04/2009 (H1N1) | WT cell | 64 |
| A/Switzerland/9715293/2013 (H3N2) | WT Cell | 32 *(GPRBC-/+ oselt 32/8)* |
| B/Brisbane/60/2008 (B/Victoria lineage) | WT Egg | 512 |
| B/Texas/02/2013 (B/Victoria lineage) | WT Cell | 512 |

TRBCs, turkey red blood cells

Virus strains are high growth reassortant or WT viruses corresponding to strains used in the 2015/16 northern hemisphere vaccination campaign.
